# Hindbrain neuropore tissue geometry determines asymmetric cell-mediated closure dynamics in mouse embryos

**DOI:** 10.1073/pnas.2023163118

**Published:** 2021-05-03

**Authors:** Eirini Maniou, Michael F. Staddon, Abigail R. Marshall, Nicholas D. E. Greene, Andrew J. Copp, Shiladitya Banerjee, Gabriel L. Galea

**Affiliations:** ^a^Department of Developmental Biology and Cancer Researching and Teaching, University College London Great Ormond Street Institute of Child Health, WC1N 1EH London, United Kingdom;; ^b^Department of Physics and Astronomy, University College London, WC1E 6BT London, United Kingdom;; ^c^Department of Physics, Carnegie Mellon University, Pittsburgh, PA 15213;; ^d^Department of Comparative Bioveterinary Sciences, Royal Veterinary College, NW1 0TU London, United Kingdom

**Keywords:** neural tube, hindbrain neuropore, mouse, biomechanics, physical modeling

## Abstract

Gap closure is a common morphogenetic process. In mammals, failure to close the embryonic hindbrain neuropore (HNP) gap causes fatal anencephaly. We observed that surface ectoderm cells surrounding the mouse HNP assemble high-tension actomyosin purse strings at their leading edge and establish the initial contacts across the embryonic midline. Fibronectin and laminin are present, and tensin 1 accumulates in focal adhesion-like puncta at this leading edge. The HNP gap closes asymmetrically, faster from its rostral than caudal end, while maintaining an elongated aspect ratio. Cell-based physical modeling identifies two closure mechanisms sufficient to account for tissue-level HNP closure dynamics: purse-string contraction and directional cell motion implemented through active crawling. Combining both closure mechanisms hastens gap closure and produces a constant rate of gap shortening. Purse-string contraction reduces, whereas crawling increases gap aspect ratio, and their combination maintains it. Closure rate asymmetry can be explained by asymmetric embryo tissue geometry, namely a narrower rostral gap apex, whereas biomechanical tension inferred from laser ablation is equivalent at the gaps’ rostral and caudal closure points. At the cellular level, the physical model predicts rearrangements of cells at the HNP rostral and caudal extremes as the gap shortens. These behaviors are reproducibly live imaged in mouse embryos. Thus, mammalian embryos coordinate cellular- and tissue-level mechanics to achieve this critical gap closure event.

## Significance

Failure to biomechanically close the embryonic neural tube in the developing brain causes fatal anencephaly. Despite their clinical importance, which cellular force-generating mechanisms close the neural tube remains poorly understood. This interdisciplinary study combines morphometric analysis, mouse embryo live imaging, and in silico modeling to formally identify cellular behaviors which complete midbrain/hindbrain closure. Two cellular force-generating behaviors not previously appreciated to act in this context are identified: contractility of supracellular actomyosin purse strings around the gap and directional movement of cells toward the gap. Both these mechanisms are required to describe gap closure, and their resulting dynamics are substantially impacted by morphogenetically imposed tissue geometry. This work provides a broadly applicable biophysical framework underlying fatal failures of midbrain/hindbrain closure.

Closure of embryonic tissue gaps is a common morphogenetic process critical to the formation of structures, including the eyelids (1), palate (2), body wall (3), and neural tube (NT) (4). The process of NT closure has long served as a paradigm of morphogenesis and remains clinically relevant today. Failure of NT closure causes severe neurodevelopmental defects in around 0.1% of human pregnancies globally (5, 6). Despite their clinical importance, the cellular force-generating mechanisms which deform embryonic tissues to close the NT are poorly understood.

NT closure starts with V-shaped bending of the flat neural plate at the hindbrain–cervical boundary, producing lateral neural folds that meet at the dorsal midline (4). The point at which the neural fold tips first meet is called Closure 1. Without Closure 1, the hindbrain and spinal NT remain open, producing craniorachischisis (4). Absence of Closure 1 formation is characteristic of homozygous mutations in core planar cell polarity components such as *Vangl2* (7–10). Soon after Closure 1 forms, a distinct elevation and midline apposition process at the midbrain–forebrain boundary establishes Closure 2 in mice. The resulting gap of open NT between Closure 1 caudally and Closure 2 rostrally is called the hindbrain neuropore (HNP) (4). Midline fusion points, referred to as “zippering” points, form at each of these closure sites and progress toward each other, completing closure when they meet. In the context of NT biology, the term zippering is used to denote tissue-level propagation of closure from a preexisting contact point, as distinct from a “buttoning” process whereby multiple midline contacts form simultaneously (11–13). Here, the zippering point which progresses rostrally from Closure 1 will be referred to as C1z and that which progresses caudally from Closure 2 as C2z to distinguish these points from the formation of the Closure points themselves, which is not studied here.

Failure to form or close the HNP produces the fatal defect exencephaly/anencephaly. At the cellular level, there remain many unanswered questions about how HNP closure is achieved. These include the relative importance of individual cell behaviors to achieving closure and how these are collectively integrated at the tissue scale. Closure rate dynamics are hypothesized to modulate anencephaly risk by determining whether it completes before a developmental “deadline,” after which tissue-level changes preclude closure (14).

Our recent studies of NT closure in the presumptive spinal region establish that it is a biomechanical event involving both the neuroepithelium and nonneural surface ectoderm (15, 16). Spinal surface ectoderm cells assemble high-tension actomyosin cables which border the region yet to close (15, 17), but whether similar cables are present in the HNP is unknown. Genetic and live-imaging evidence also implicates the surface ectoderm in HNP closure. Deletion of surface ectoderm genes such as Par1/2 (18) and Grhl3 (19, 20) produces exencephaly. More directly, surface ectoderm cells produce delicate cellular ruffles, which extend across the embryonic midline to meet their contralateral equivalents (12, 21, 22). These projections are proposed to mediate midline fusion (23). Detailed analysis of equivalent protrusions in the spinal NT found they are genetically controlled by actomyosin cytoskeletal Rho-GTPase regulators (24).

Genetic deletion or pharmacological inhibition of actomyosin and its regulators commonly produces exencephaly in mice (25–28). Actomyosin contractility, turnover, and cytoskeletal assembly determine tissue tension in neurulation-stage vertebrate embryos (15, 29). This contractile network is interlinked between multiple cells in epithelia through direct anchoring to adherens junctions. Supracellular actomyosin sheets or cables allow cells to act collectively in deforming their tissues (30, 31). In the studies presented here, we provide biophysical characterization of closure of the HNP, subsequent to the neural fold elevation and apposition events which initially form it. We identify and biomechanically characterize surface ectoderm actomyosin purse strings which contribute to HNP closure and test their contributions using cell-based physical modeling corroborated by mouse embryo live imaging. We find that a combination of purse-string contractility and directional movement of the surface ectoderm cells, simulated through cell crawling, are both required to account for HNP closure dynamics, but tissue geometry constrains rostrally directed zippering, producing a rate asymmetry in cell-mediated closure.

## Results

### Surface Ectoderm Purse Strings Encircle the Closing Mammalian HNP.

**Fig. 1.**
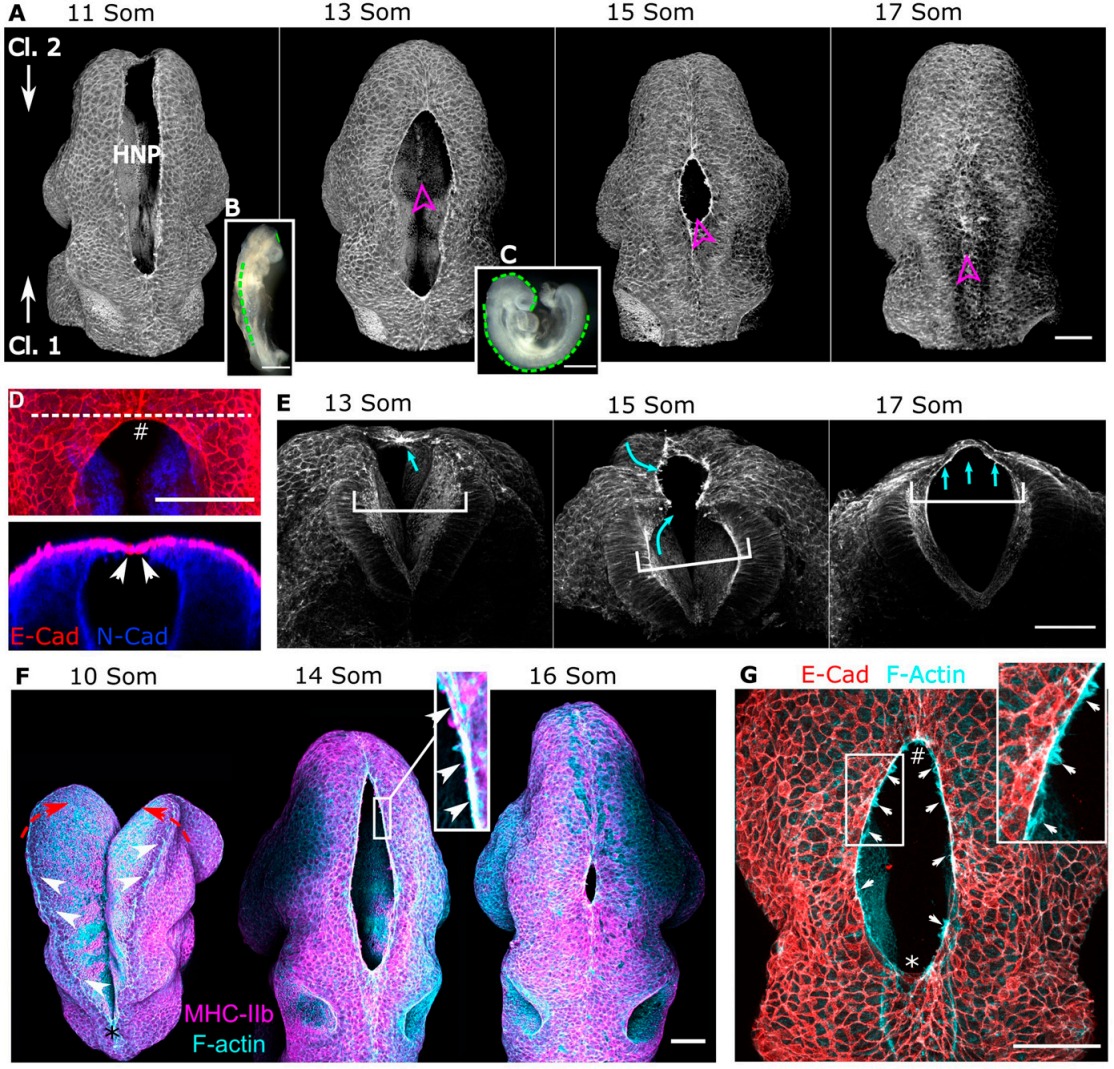
Surface ectoderm purse strings encircle the mammalian HNP. Representative whole-mount images of the developing cranial region in wild-type C57BL/6J mouse embryos. Throughout, zippering progression from Closure 2 is at the top, and progression from Closure 1 is at the bottom of the image. (*A*) Three-dimensional reconstructions of phalloidin-stained embryos illustrating the progression of HNP closure. Somite (Som) stages are as indicated. Magenta arrowheads indicate the direction of sectioning of the same embryos shown in *E*. (*B* and *C*) Stereoscope images of 11 Som (*B*) and 15 Som (*C*) stage embryo. The dashed green lines indicate regions where the NT is closed. (*D*, *Top*) Representative dorsal view of the C2z with the neuroepithelium stained with *N*-cadherin and surface ectoderm with E-cadherin. (*Bottom*) An optical reslice along the dashed line is shown below; arrowheads indicate the first point of contact, which is between surface ectoderm cells. (*E*) Transverse views into the same embryos indicated in *A*. White brackets indicate the distance between the lateral walls of the NT underlying the HNP. Cyan arrows indicate medial extension of a layer of cells, forming a thin closed layer by the 17 Som stage. (*F*) Dorsal views of representative embryos before (10 Som, * indicates the C1z) and after HNP formation. Actomyosin cable-like enrichments (arrows) are detected along the open neural folds. Red arrows indicate the necessary elevation of the neural folds, which precedes HNP formation. (*Inset*) Actin and myosin colocalization in the encircling cable (arrows). (*G*) Colocalization of cable F-actin with the surface ectoderm marker E-cadherin. (*Inset*) Membrane F-actin–rich ruffles (arrows) which appear to extend from the actomyosin cables.

The mouse HNP forms when the neural folds elevate and come into apposition at Closure 1 caudally and Closure 2 rostrally, closing progressively over an ∼12 h period (Fig. 1 *A*–*C*). The first points of contract during HNP closure are established by E-cadherin expressing surface ectoderm cells (Fig. 1*D*). Underlying this lie the elevated lateral walls of the NT, which remain > 100 µm apart (Fig. 1*E*). Beyond the dorsal tips of these, a thin tissue layer led by the surface ectoderm followed by neuroepithelial cells is suspended dorsally over the HNP gap (Fig. 1 *D* and *E*, 15 somites). In embryos which recently completed HNP closure, the roof of the NT is composed of a thin cell bilayer suspended over fluid-filled space, as the lateral walls of the NT remain far apart (Fig. 1*E*, 17 somites).

Throughout this closure process, actomyosin purse strings demarcate the HNP rim (Fig. 1*F*). They line the neural fold tips at the boundary between the surface ectoderm and neuroepithelium (Fig. 1*F*). High-resolution imaging demonstrates cable colocalization with the surface ectoderm marker E-cadherin, thereby showing their presence in surface ectoderm cells (Fig. 1*G*). F-actin–rich membrane ruffles are also visible along the length of the HNP (Fig. 1*G*), emanating from the cable-producing cell borders. These ruffles are reminiscent of the membrane protrusions characteristic of migrating cells (32, 33). High-resolution visualization of F-actin in surface ectoderm cells at the HNP rim, denoted Row 1, shows dense enrichments at their gap-facing end and stress fiber-like linear arrangements oblique to the gap rim (*SI Appendix*, Fig. S1 *A* and *B*). These linear arrangements can also be seen in trailing cells not engaged in the HNP gap, although actomyosin intensity is lower in trailing Row 2 and 3 cells then leading-edge Row 1 cells (*SI Appendix*, Fig. S1 *C*–*E*).

Both laminin and fibronectin are present under these leading cells (*SI Appendix*, Fig. S2 *A* and *B*). These extracellular matrix (ECM) proteins appear sparser close to the HNP rim, potentially suggesting their ongoing assembly, but are nonetheless present as adhesion substrates at the encircling F-actin rim (*SI Appendix*, Fig. S2 *A* and *B*). Fibronectin fibril assembly involves stabilization on integrins, which recruit the nascent cell–ECM adhesion protein tensin 1 (TNS1) (34). TNS1 immunolocalization in surface ectoderm cells shows focal puncta reminiscent of focal adhesions as well as structures resembling basal bodies (*SI Appendix*, Fig. S2 *C* and *D*), where focal adhesion components have previously been shown to localize (35). AiryScan superresolution imaging of the HNP rim shows abundant TNS1 localization basally, abutting the F-actin cables (*SI Appendix*, Fig. S2 *E* and *F*).

### HNP Closure Progresses Faster Caudally Than Rostrally.

**Fig. 2.**
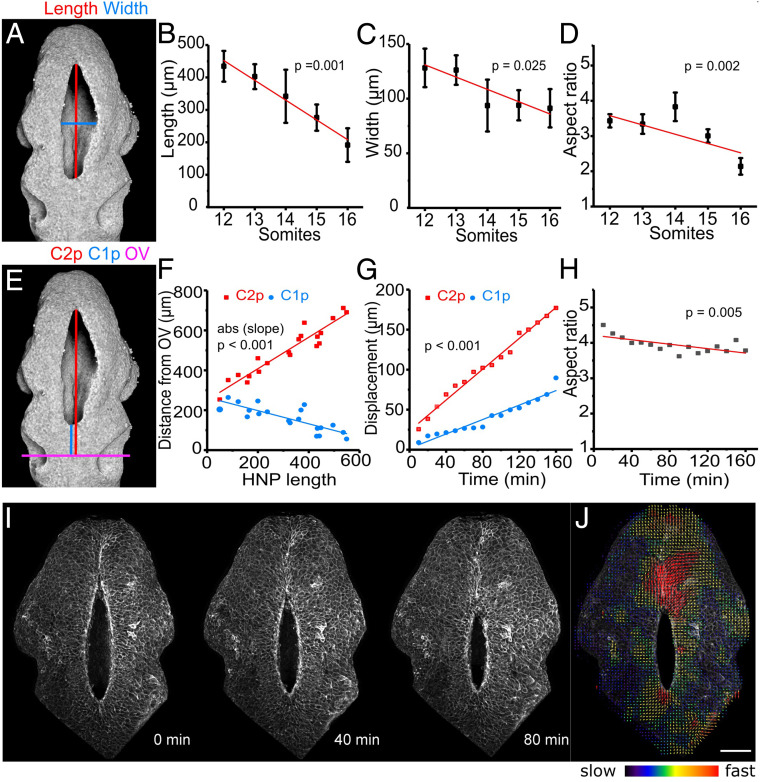
HNP closure progresses faster in the caudal than in the rostral direction, while HNP shape remains elliptical. (*A*) Three-dimensional reconstruction of a representative early (14 somite) HNP reflection image. The red and blue lines indicate HNP length and width, respectively. (*B*–*D*) Quantification of HNP length (*B*), width (*C*), and aspect ratio (*D*) in fixed embryos at the indicated somite stages (*n* = 40). The HNP shortens and narrows as it closes, but it maintains an aspect ratio greater than 2. In all cases, the slope is significantly different from 0 (*P* < 0.05, ANOVA). (*E*) Same image as in *A*. The magenta line shows the mid otic vesicle (OV) level. The red and blue lines show the distance of C2z progression (C2p) and C1z progression (C1p) from mid OV, respectively. (*F*) Quantification of the distances shown in *E* against HNP length in fixed embryos (*n* = 20). The absolute slopes of the regression lines are significantly different from one another. *P* < 0.001, F-test. (*G*) Quantification of C2p and C1p (displacement from T = 0, not the mid-OV level) over time in a live-imaged embryo (shown in *I*). The slopes of the regression lines are significantly different from one another. *P* < 0.001, F-test. (*H*) Quantification of HNP aspect ratio over time in the same live-imaged embryo (*I*). The HNP maintains a highly elliptical shape throughout closure. *P* indicates the slope is significantly different from 0. (*I*) Snapshots of a live-imaged mTmG mouse embryo at the time points indicated (14 somites at first frame). (*J*) Particle image velocimetry illustrating increased cell speed at C1z and C2z. (Scale bar, 100 μm.)

We next documented tissue-level dynamics of HNP closure using a series of fixed embryos and live imaging in whole-embryo culture. During HNP closure, both the length and width of the open region decrease with advancing somite stage (Fig. 2 *A*–*C*). The resulting shape of the HNP is initially highly elongated, with a length/width aspect ratio exceeding 3. This aspect ratio decreases slightly but maintains an elongated shape throughout the closure period (aspect ratio > 2, Fig. 2*D*). Over this time, the C1z moves rostrally, whereas the equivalent point from C2z moves caudally. Their relative contribution to closure was inferred by calculating the distance between each zippering point and the developing otic pits, used as normalization landmarks in fixed embryos (Fig. 2*E*). HNP zippering by the C2z progresses ∼400 µm within 12 h (2 h per somite, closure over somite stages 12 to 17), compared with ∼200 µm by C1z on average (Fig. 2 *E* and *F*).

These two features of HNP closure dynamics, namely faster rostral to caudal progression of closure while maintaining an elongated aspect ratio, are also observed during live imaging (Fig. 2 *G*–*I* and Movie S1). Particle image velocimetry analysis suggests wide-ranging cell displacement around the HNP rim, with the highest velocities at the zippering points (Fig. 2*J*). The underlying lateral walls of the NT remain a consistent distance apart during live imaging, while a thin cell layer led by surface ectoderm ruffles extends medially (*SI Appendix*, Fig. S3 *A* and *B*).

Asymmetry in the rate of closure means progression by C2z is responsible for forming a larger proportion of the HNP-derived roof plate and raises the possibility that the two closure points act independently of each other. We next tested closure point interdependence directly using a transgenic model which lacks Closure 1.

### Progression of HNP Closure from Closure 2 Is Independent of Closure 1.

**Fig. 3.**
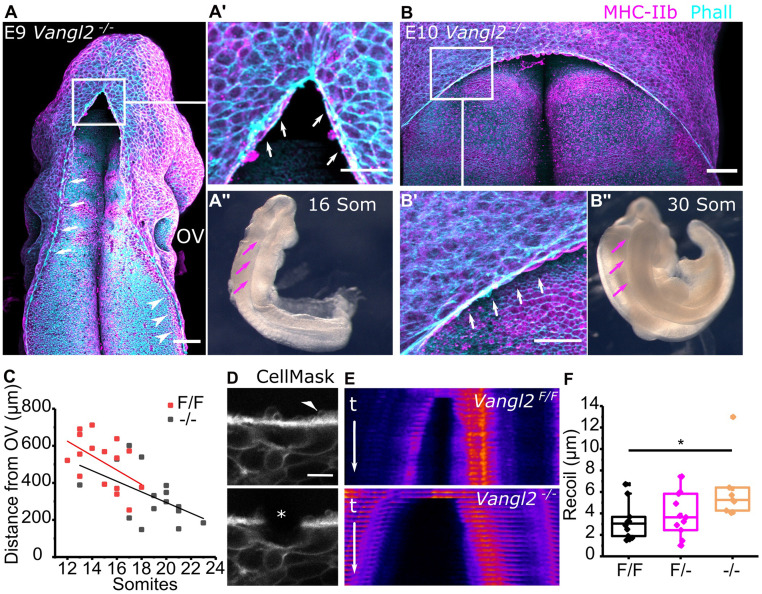
Progression of HNP closure from Closure 2 is independent of Closure 1. (*A*–*A’’*) Representative whole-mount staining and corresponding bright-field image of an early *Vangl2*^*−/−*^ embryo (16 somites [Som]). Annotated are the actomyosin cables and associated ruffles extending along the open hindbrain (white arrows in *A* and *A’*) and spinal region (white arrowheads in *A*). Magenta arrows indicate the open neural folds in bright field (*A’’*). (Scale bar, 100 μm.) (*B*–*B’’*) Representative whole-mount staining and corresponding bright-field image of a late *Vangl2*^*−/−*^ embryo (30 Som). Annotated are the actomyosin cables and associated ruffles extending along the open hindbrain (white arrows in *B’*). Magenta arrows indicate the open neural folds in bright field (*B’’*). (Scale bar, 100 μm.) (*C*) Quantification of the distance between the C2z and the mid otic vesicle (OV) level in *Vangl2*^*−/−*^ and *Vangl2*^*F/F*^ (control) embryos at the indicated somite stages. The OV level was defined as in Fig. 2*E*. (*D*) Representative laser ablation of cable-associated cell borders near the C2z. The asterisk shows the ablated border. The white arrowhead points at membrane ruffles, which colocalize with the cable (Fig. 1*G*). (Scale bar, 10 μm.) (*E*) Representative kymographs of cable ablations in *Vangl2*^*F/F*^ and *Vangl2*^*−/−*^ embryos. t indicates timeframes postablation (<1 s/timeframe) and is the same for both kymographs. (*F*) Recoil quantification after cable ablations in *Vangl2*^*F/F*^ (*n* = 10), *Vangl2*^*F/−*^ (*n* = 11), and *Vangl2*^*−/−*^ (*n* = 7) at 12 to 17 Som stages. *P* < 0.05, ANOVA with Bonferroni post hoc correction.

Zippering C2z is not impaired in embryos which lack Closure 1 because of the deletion of *Vangl2* (Fig. 3 *A*–*C*). These *Vangl2*^*−/−*^ embryos invariably develop craniorachischisis (Fig. 3 *A* and *B’’*). Nonetheless, their C2z point assembles actomyosin cables (Fig. 3 *A*, *A’*, *B*, and *B’*), and in the absence of rostral zippering from Closure 1, zippering can progress from Closure 2 to a more caudal level, at least 100 µm closer to the otic pits than their wild-type counterparts (Fig. 3*C*).

Without Closure 1, the actomyosin cables cannot form an encircling purse string but instead are present as long cables extending caudally from C2z as far as the unfused spinal neural folds (Fig. 3 *A* and *B*). Persistent myosin enrichment suggests they are contractile, despite this dramatic difference in morphology. Confirming this, actomyosin cable tension inferred from recoil after laser ablation is greater in *Vangl2*^*−/−*^ embryos than wild-type littermates (Fig. 3 *D*–*F*). Cable recoil was assessed in the comparable region adjacent to C2z at equivalent somite stages. Thus, this transgenic model of extreme HNP asymmetry demonstrates that zippering from rostral and caudal closure points is largely independent of each other.

### Closure Rate Asymmetry Does Not Arise from Local Differences in Mechanical Tension.

**Fig. 4.**
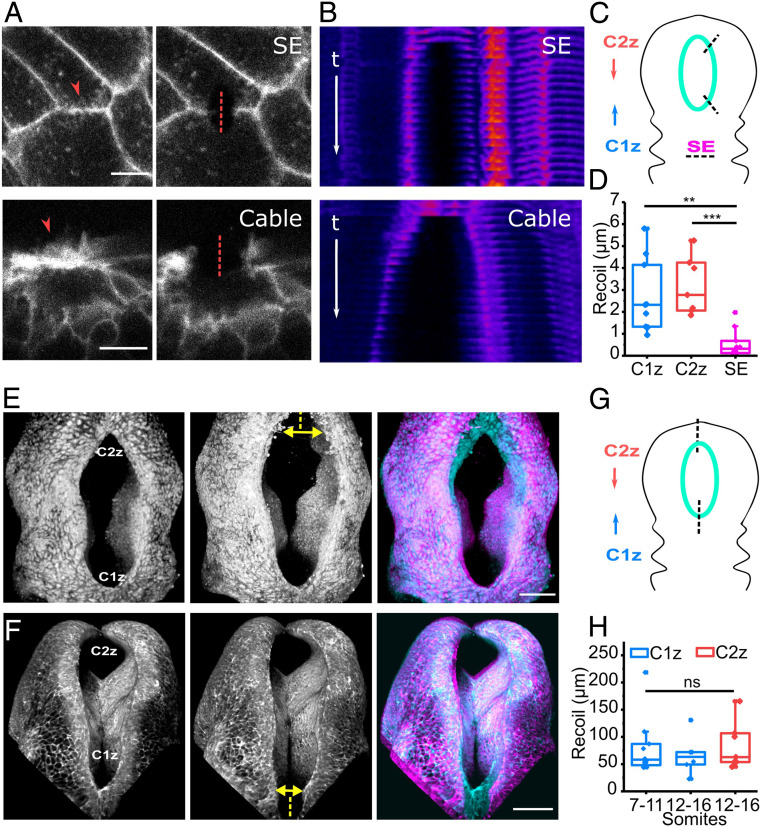
Biomechanical tension is comparable between the rostral and caudal ends of the HNP. (*A*) Representative laser ablations of surface ectoderm (SE) border (*Top*) and cable near the C2z (*Bottom*). The arrowhead points at membrane ruffles, and the dashed line shows the site of ablation. (Scale bar, 10 μm.) (*B*) Kymographs corresponding to the ablations in *A* shown in Fire lookup table. t indicates timeframes postablation (<1 s/timeframe) and is the same for both kymographs. Cell borders show faster separation from the ablation site after cable than noncable SE border ablation. (*C*) Schematic representation of positions where SE cell border and cable ablations were performed. The HNP rim is indicated in cyan. (*D*) Recoil quantification after cable ablations proximal to C1z (*n* = 9), C2z (*n* = 7), and ablations of SE borders in the closed region (*n* = 10). **P < 0.01, ***P < 0.001 ANOVA with Bonferroni post hoc correction. (*E* and *F*) Three-dimensional reconstruction of representative zippering point (tissue level) laser ablations. Ablations of C2z and C1z are shown in *E* (14 somites) and *F* (13 somites), respectively. The dashed line shows the site of ablation, and the arrow indicates lateral recoil of the neural folds. Right shows the overlay of pre- and postablation images in cyan and magenta, respectively. (Scale bar, 100 μm.) (*G*) Schematic representation of zippering point (tissue level) ablations. The HNP rim is indicated in cyan. (*H*) Recoil quantification after ablations of the C1z zipper at 7 to 11 somites (*n* = 10) and 12 to 16 somites (*n* = 6) and the C2z (*n* = 7). NS: nonsignificant, ANOVA with Bonferroni post hoc correction.

In wild-type mice, the actomyosin purse string appears to link the two closure point zippers and differential contractility might explain asymmetric closure rates. Contrary to this, we found that tension withstood by surface ectoderm cell borders engaged in the purse-string cables, inferred from recoil following laser ablation, is comparable between the rostral and caudal ends of the HNP (Fig. 4 *A*–*D*). Surface ectoderm cell borders overlaying the closed NT were ablated as comparators, demonstrating approximately fivefold lower recoil compared to cable cell borders after laser ablation (Fig. 4 *A*–*D*).

In addition to cell border ablations, tissue-level ablations were performed in independent embryos to infer mechanical tension opposing HNP closure, as we previously reported in the spinal NT (15–17, 36). Tissue-level laser ablations of either the C1z or C2z both produced lateral recoil of the neural folds, widening the HNP in the 3 min required to obtain the postablation image (Fig. 4 *E*–*H*). The underlying lateral walls of the NT also move laterally following ablation (*SI Appendix*, Fig. S4 *A*–*C*). This demonstrates that the recently fused NT is load bearing.

We next tested whether tissue-level tension changes as Closure 2 forms, converting the open cranial neural folds into an HNP. The magnitude of lateral recoil at C1z is not significantly different between early developmental stages before Closure 2 forms versus later stages when both zippering points are present (Fig. 4*H*). Recoil is also comparable when either the C1z or C2z is ablated in different embryos (Fig. 4*H*).

Thus, neither differential proclosure contractility (cable tension) nor anti-closure forces (tissue-level lateral recoil) are sufficient to explain asymmetric HNP closure dynamics.

### Combination of Medial Cell Movement and Purse-String Contraction Describes HNP Dynamics.

**Fig. 5.**
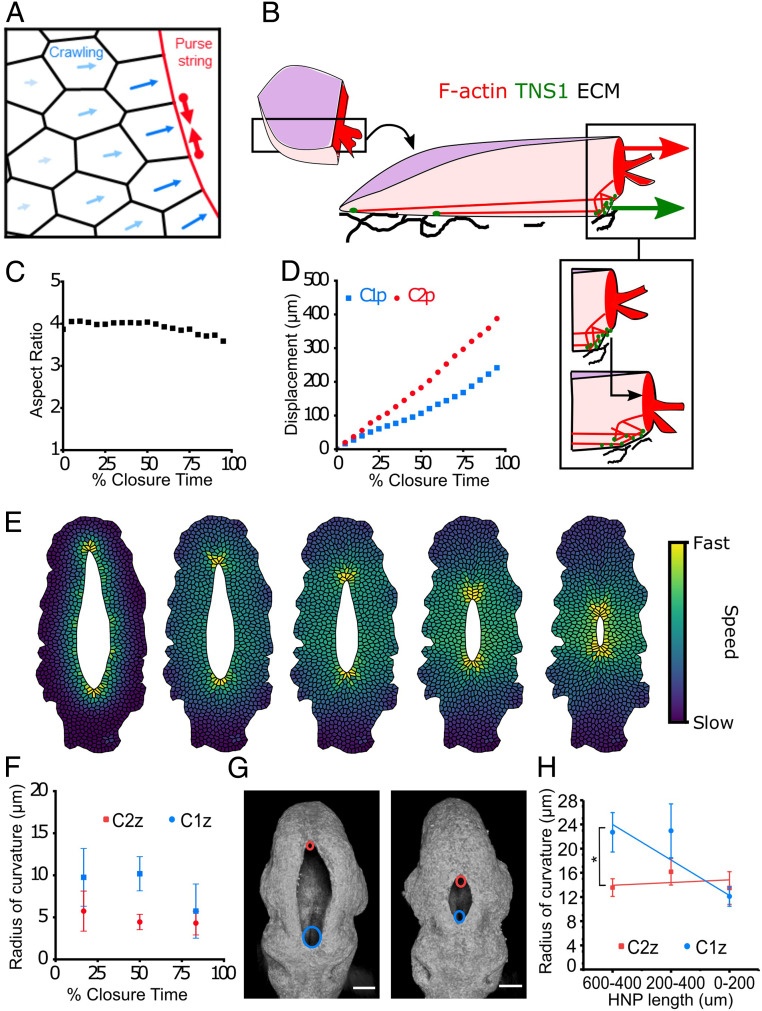
Cell-based modeling reveals that asymmetric geometry regulates closure rate asymmetry. (*A*) Schematic of the vertex model for HNP gap closure. Cell edges on the gap, highlighted in red, have an increased tension because of the assembly of actomyosin purse string. Cells actively crawl toward the gap but with less speed as distance to the gap increases (blue arrows). (*B*) Schematic representation of the proposed force-generating mechanisms employed by surface ectoderm cells at the HNP gap. These cells assemble F-actin at their leading-edge, purse-string membrane ruffles and stress fiber-like arrangements basally. The early focal adhesion marker TNS1 localizes in basal puncta and is enriched directly behind the purse string. Gap-directed forces are produced by purse-string contractility (red arrow) and displacement onto newly assembled ECM (“crawling,” green arrow). The bottom inset illustrates how purse-string contraction and crawling are proposed to occur. As the purse string pulls the leading edge forward (black arrow), TNS1-positive adhesions accumulate behind it, where they are able to bind to and facilitate the assembly of ECM. (*C*) Gap aspect ratio against percentage closure time, as outputs of the model combining purse-string contraction and cell crawling. (*D*) Progression of C1z and C2z against percentage closure time, as outputs of the model. (*E*) Time course of simulated gap closure, at 10, 30, 50, 70, and 90% of closure time, from left to right. Cell color indicates cell speed. (*F*) Simulation mean radius of curvature at the C1z and C2z. Data are binned into the nearest tertile of closure. Error bars represent SD within bins (*n* = 56). (*G*) Three-dimensional reconstructions of representative early (14 somites, *Left*) and late (16 somites, *Right*) HNPs. A circle was fitted at each zippering point to calculate the radius of curvature. Cyan and red circles annotate C1z and C2z, respectively. (*H*) Experimental mean radius of curvature at the C1z and C2z plotted against HNP length. Error bars represent SE (*n* = 14 to 17 for each bin). *P* < 0.05, paired *t* test.

To better understand the mechanistic origin of HNP closure dynamics, and specifically the differential closure rates between C1z and C2z, we developed a vertex-based mechanical model of surface ectoderm cells. In two-dimensional vertex models, as implemented here, a network of edges represents cell–cell junctions, and the polygons represent the apical surfaces of the cells (Fig. 5*A*) (37, 38). Note that this model is only intended to describe closure of the thin cell layer, which extends medially during HNP closure, not the elevation of the underlying neural folds and their apposition at Closure 2, which initially forms the HNP. Each cell has a mechanical energy composed of area elasticity, cytoskeletal contractility, and interfacial tension at the cell–cell junctions. Cell edges lining the HNP gap have an increased tension due to the assembly of the contractile actomyosin purse string, which generates a driving force for gap closure (Fig. 5*A*). Potential models were iteratively tested for their ability to replicate the observed tissue-level HNP closure dynamics, namely an asymmetric closure rate, while maintaining an elongated aspect ratio over long timescales. The initial gap geometry and cell centroids were based on those of a representative HNP in a 12-somite stage embryo.

Purse-string contractility is nominally sufficient to achieve gap closure but produces a progressively more circular gap because the zippering points move rapidly toward each other with very little lateral motion until gap aspect ratio drops below the range observed in vivo (*SI Appendix*, Fig. S5 *A*–*D* and Movie S2). To maintain an elongated gap shape, we included active forces, which extend the surface ectoderm medially. These were implemented through cell crawling throughout the bulk of the cell, assumed to occur via adhesions onto newly assembled ECM directly behind their leading edge (Fig. 5*B*). Cell crawling alone is also nominally sufficient to close the gap, but the gap width and length close at the same speed, increasing gap aspect ratio over time before both sides meet laterally (*SI Appendix*, Fig. S6 *A*–*D* and Movie S2). Simulating HNP closure from an empirically determined initial geometry with a combination of purse-string and crawling mechanisms maintains gap aspect ratio over long timeframes (Fig. 5*C*), closely recapitulating the pattern seen in vivo (Fig. 2*H*).

Varying the speed of cell crawling relative to the default model demonstrates that increasing crawl speeds increases overall closure rates (*SI Appendix*, Fig. S6*G*) and leads to the maintenance of gap aspect ratio for longer times (*SI Appendix*, Fig. S6*H*). Taken together, these simulations suggest that both purse-string contraction and active cell crawling can contribute to the rate of HNP closure, and their combination maintains gap aspect ratio over time. We find that asymmetry between rostral and caudal closure rates is greater when purse-string contraction is the only driving force (*SI Appendix*, Fig. S5*B*), is present when cell crawling is the only closing force (*SI Appendix*, Fig. S6*B*), and continues to be observed when both purse-string contraction and cell crawling are implemented (Fig. 5*D*). This asymmetry in closure rate arises despite equal cable tension or crawling forces at C1z and C2z.

Recent work has suggested that gap geometry plays an important role in regulating the dynamics of wound closure, such that the rate of purse-string–driven closure is proportional to the gap curvature (39–41). Indeed, the HNP geometry substantially differs between the rostral and caudal regions (Figs. 1 *A* and *G* and 2*A*), motivating us to investigate the relationship between closure rate asymmetry and HNP geometry.

### Asymmetric Tissue Geometry Produces Closure Rate Asymmetry.

Simulated closure of an arithmetically elliptical gap, rather than empirically determined HNP geometry, produces equivalent rates of closure from the two extremes (*SI Appendix*, Fig. S7*A*). For a rate asymmetry to exist, there must be some difference between the two closure points in either the driving forces or the resistive forces arising from the surrounding tissue. At early stages, there are fewer cells in the plane of the gap in the recently closed region rostral to C2z than there are in the closed region caudal to C1z (*SI Appendix*, Fig. S8). Closure of an elliptical gap on biologically realistic boundary conditions progresses only slightly faster from the end with fewer cells (*SI Appendix*, Fig. S7*B*). Exaggerating differences in boundary conditions increases closure rate asymmetry (*SI Appendix*, Fig. S7*C*). Thus, an ellipse can close asymmetrically when fewer cells need to be rearranged at one apex than the other, but this effect is insufficient to explain the observed closure rate asymmetry under biologically relevant conditions.

An additional geometric feature incorporated in the simulation is that in early embryos with long HNPs, the C1z has a greater radius of curvature than that at the C2z (Fig. 5 *F*–*H*). In both the simulation (Fig. 5*F*) and in vivo (Fig. 5 *G* and *H*), C2z maintains a low radius of curvature (more acute angle), while C1z’s radius of curvature decreases over developmental time. Since the purse-string acts as a cable under tension, the resulting force will be inversely proportional to the radius of curvature, resulting in a larger net force and faster closure at C2z (*SI Appendix*, Fig. S5*B*). Consequently, simulated purse-string–driven closure displays gap length-dependent dynamics: It slows down as the HNP becomes rounder, before speeding up again when the gap is very small (*SI Appendix*, Fig. S9).

The rate of shortening is more constant when crawling is implemented alone or in addition to purse-string contraction (*SI Appendix*, Fig. S9). A constant shortening rate is more consistent with the overtly linear relationship between HNP length and somite stage observed in vivo (Fig. 2*B*). Closure rate remains faster from C2z when cell crawling is the only driving force (*SI Appendix*, Fig. S6*B*). This is because the gap is narrower near the C2z, so the edges meet sooner. Incorporating this experimentally determined gap geometry in the final model reproduces the asymmetry in closure rates observed in vivo (Fig. 5 *D* and *E*).

### Surface Ectoderm Cells Display HNP Gap-Directed Displacement In Vivo.

**Fig. 6.**
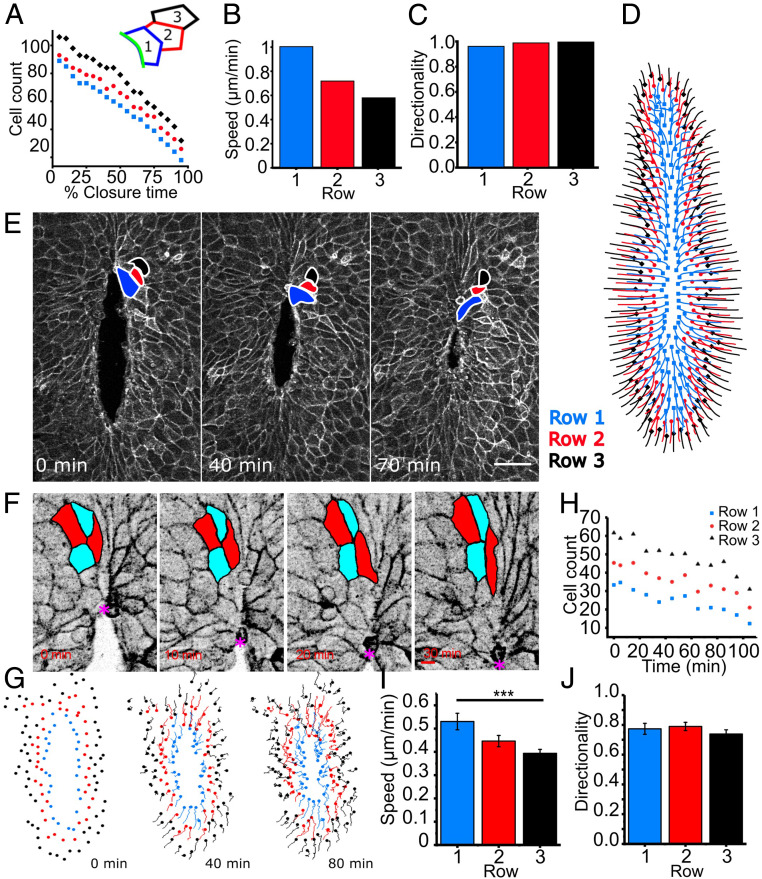
Directional migration of surface ectoderm cells toward the HNP gap. Data in *A*–*D* are from simulated gap closure achieved by combined purse-string constriction and cell crawling, whereas *E*–*I* are from live-imaged embryos. (*A*) Cell count in the first three rows around the gap against closure time. The inset illustrates the three cell rows analyzed. Row 1 cells (blue) assemble the cables (green line) at the HNP rim. Row 2 cells (red) contact Row 1 without being engaged in the cable. Row 3 (black) contact Row 2 cells. (*B* and *C*) Mean speed (*B*) and mean directionality (*C*) per row. (*D*) Cell center trajectories over the course of simulated closure. Color indicates initial cell row. (*E*) Snapshots of a live-imaged, 15-somite mTmG mouse embryo, showing progression of HNP closure. Row 1 to 3 cells are annotated. (Scale bar, 100 μm.) (*F*) Live imaging of cells rostral to Closure 2 (*), showing a T1 transition as the two red cells become separated by the two cyan cells (same embryo as *E*, inverted gray lookup table). (Scale bar, 10 μm.) (*G*) Tracks of individual cells at the first three rows around the HNP shown in *E*, illustrating directional cell displacement during live imaging. (*H*) Number of cells per row over time showing equivalent rates of reduction in row occupancy. (*I*) Mean cell speed per row for the first 25 min, showing Row 1 cells underwent faster displacement than Rows 2 or 3 in this embryo. *P* < 0.001, one-way ANOVA with Bonferroni post hoc correction. (*J*) Mean cell directionality per row for the first 25 min, showing equivalence between rows.

Having developed an in silico model which meaningfully recapitulates tissue-level dynamics, we used it to predict the underlying cell-level dynamics around the HNP rim. In particular, we investigated the dynamics of three rows of cells around the HNP, with Row 1 being the cells which form the actomyosin purse strings (Fig. 6*A*, *Inset*). The three rows describe concentric rings with progressively more cells in each row. During simulated HNP closure, the number of cells in each row decreases gradually, with row occupancy decreasing at the same rate in each row (Fig. 6*A*). Cells nearest to the gap move with greater speed during closure (Fig. 6*B*, initial speeds defined in the model) but have comparable directionality (defined as Euclidian distance divided by total distance traveled) compared to the surrounding rows (Fig. 6 *C* and *D*).

With only cell crawling, or only purse-string contraction, the speed is highest, and directionality lower, in the cells closest to the gap (purse string *SI Appendix*, Fig. S5 *E* and *F*; crawling *SI Appendix*, Fig. S6 *E* and *F*). However, the directionality of Row 1 cell displacement is lower (< −0.7 Euclidian/accumulated distance) when purse-string–mediated closure is implemented without crawling (*SI Appendix*, Fig. S6*F*), because cells display large curving trajectories (*SI Appendix*, Fig. S6*C*). Furthermore, when closure is only achieved by purse-string constriction, the cells along the lateral rims of the HNP do not displace until gap curvature drops below the range observed in vivo (*SI Appendix*, Fig. S5*D* and Movie S2). This is because the speed of cell motion in this model is proportional to curvature, which is very low along the lateral borders of the HNP when the aspect ratio is >2. As purse-string constriction pulls Row 1 cells toward the gap, they in turn pull Row 2 cells and so on.

We developed subcellular live-imaging capability in order to visualize surface ectoderm rearrangement around the closing HNP in vivo (Fig. 6 *E*–*G* and Movie S3). We observed that Row 1 cells contribute to the HNP rim until they reach the zippering point, after which they release their connection to the rim (Fig. 6*E*) and undergo classical T1 transitions as they make new partners with cells formerly in Rows 2 and 3 (Fig. 6*F*). Manual tracking of surface ectoderm cells in the first three rows confirms cells displace toward the HNP gap (Fig. 6*G* and Movie S4). Note that cell displacement is also observed along the lateral, relatively flat borders of the HNP (Fig. 6*G* and Movie S4).

As predicted by our vertex model, the rates at which cells leave each row is equivalent between rows (Fig. 6*H*, representative of four out of four independently live-imaged embryos). Cell displacement parameters were analyzed over the first 25 min of live imaging to reduce reanalysis of cells which switch rows. Over this period, cells in Row 1 had a significantly higher speed than Row 3 in two out of four embryos (representative in Fig. 6*I*) and an equivalent speed in the remaining two out of four embryos. Row 1 cells had equivalent (three out of four embryos, Fig. 6*J*) or significantly higher (one out of four embryos) directionality than Row 3 cells. Directionality is invariably underestimated in manually tracked data because of “juddering” displacement paths caused by changes in cell shape between time points. Nonetheless, Row 1 directionality is > 0.7 Euclidian/accumulated distance in all four independent embryos (*SI Appendix*, Fig. S5*F*). Taken together, live imaging confirms that highly directional surface ectoderm cell displacement, consistent with crawling toward the gap, contributes to HNP closure.

## Discussion

The interdisciplinary studies described here establish a conceptual biophysical framework through which disruption, or enhancement, of HNP closure can be assessed. Tissue geometry, directional surface ectoderm displacement, and actomyosin purse-string contractility emerge as necessary parameters sufficient to describe the simulated dynamics of HNP closure. Each of these three parameters will be responsive to a large number of genes and signaling cascades whose enhancement may promote timely closure and disruption may impede closure. Simulation demonstrates both purse-string contractility and directional movement accelerate closure from Closure 2, because tissue geometry constrains progression from Closure 1. This means a greater proportion of the mouse HNP roof plate is produced by zippering from Closure 2. Moreover, our model captures the directional dynamics of surface ectoderm cells surrounding the gap visualized by live imaging.

Model systems used to study gap closure include in vitro wounds (42–45), *Drosophila* dorsal closure (46, 47), embryonic wound healing (48, 49), nematode ventral closure (50), and *Xenopus* blastopore closure (51, 52). Recurring mechanisms include the formation of contractile actomyosin cables at tissue interfaces, cell migration, and partner exchange across the gap midline (47, 53, 54). However, differences in gap geometry and cell types involved preclude direct extrapolation of mechanisms identified in other systems to closure of the mammalian NT.

A previously described mechanism of HNP closure is the establishment of cell contacts across the midline by surface ectoderm cell protrusions (22, 23). We now find that these protrusions appear to emanate from actomyosin purse strings surrounding the HNP rim. ECM components can provide an adhesion substrate at the HNP rim, where we observe accumulation of the early focal adhesion marker TNS1. Actomyosin purse strings are robust morphogenetic tools, which couple cell-level control of morphogenesis to tissue-level deformation (17, 55, 56). The requirement for actomyosin purse strings in gap closure has recently been questioned in *Drosophila* because dorsal closure completes successfully in embryos lacking high-tension purse strings (57, 58). However, there are substantial differences between these systems: The early HNP is approximately double in length and takes three times as long to close as the *Drosophila* dorsal gap. Dorsal closure is slower in fly embryos which lack purse strings (57, 58), suggesting that one of the roles of these structures in the HNP could be to ensure closure completes before the previously suggested (14) developmental deadline.

The importance of seemingly subtle tissue geometric properties in determining HNP closure rate have not, to our knowledge, previously been appreciated. The HNP geometry from which our analyses begin is established after elevation and apposition of the underlying neural folds, which form Closure 1 and Closure 2. As documented here, the lateral walls of the NT do not continue to come into apposition as the HNP gap closes. The inward closure force generated by the purse string is greater in regions of higher rim curvature (40), increasing cell speeds at the C2z point. In addition, cells have less distance to cross at the narrow Closure 2 compared to C1z. Why the C1z point is wider than that at Closure 2 in the early HNP is unclear. Potential explanations include very different mechanisms underlying the initial formation of these closure points and whole-embryo deformation during axial rotation after Closure 1 forms (59). Intriguingly, the initial position at which Closure 2 forms varies between genetically wild-type mouse strains (60), suggesting a degree of functional redundancy. Morphogenetic redundancy, or compensation, is demonstrated in this study by the ability of the C2z point to proceed further caudally in the absence of Closure 1.

The embryos with craniorachischisis studied here also demonstrate that the assembly of high-tension actomyosin supracellular enrichments does not require Closure 1 or expression of *Vangl2*. This is consistent with a previous report that *Vangl2* deletion does not impair wound healing in the fetal epidermis (61). Increased actomyosin purse-string tension in *Vangl2*^*−/−*^ embryos compared with littermate controls could either indicate *Vangl2* normally suppresses contractility or, more likely, may be secondary to tissue-level structural differences. It is well established that mechanical tensions trigger mechanochemical feedback mechanisms, which increase nonmuscle myosin recruitment (62) and adherens junction stability (63).

Establishment of E-cadherin adherens junctions between surface ectoderm cells on opposite sides of the embryonic midline allows advancement of the zippering points. The zippering dynamics observed during live-imaged HNP closure in this work is different from what has been described in other closure processes. Individual surface ectoderm cells at the zipper appear to follow this point, often persisting at the leading edge for over one cell length. In contrast, live imaging of *Ciona* NT closure shows shrinkage of cell junctions ahead of the zipper and direct matching of cells across the embryonic midline (64). Although “buttoning” had previously been suggested to close the HNP based on lower-resolution live imaging (13), no evidence of buttoning protrusions are observed in freshly dissected embryos fixed directly after removal from the uterus nor are they visible in the high-resolution live imaging provided here.

Previous live imaging of mouse HNP closure also demonstrated displacement of cell nuclei at the leading edge of the gap (65). The first row of surface ectoderm cells extends protrusions toward the neuropore, similarly to the closure of large gaps in wound healing (66, 67). Stochastic lamellipodia-driven migration of a small number of leader cells results in “rough” edges of the closing gap (54). In contrast, the closing HNP gap has “smooth” edges, presumably due to coordinated actomyosin cable constriction in Row 1 cells. It remains to be established how directionality is inferred in Row 1 cells and how these cells remodel their cell–cell and cell–ECM attachments to achieve gap-oriented displacement.

In summary, the biophysical framework presented here begins deconstructing cellular mechanisms of HNP closure from morphometric measurements. Our findings extend the generalizability of core proclosure modules beyond the size and timescales commonly studied in simpler organisms. Their concurrence encourages generalization to other closure events of both scientific and clinical importance.

## Materials and Methods

### Animal Procedures.

Studies were performed under the regulation of the UK Animals (Scientific Procedures) Act 1986 and the Medical Research Council’s Responsibility in the Use of Animals for Medical Research (1993). C57BL/6 mice were bred in house and used as plug stock from 8 wk of age. Mice were mated overnight, and the next morning a plug was found and considered E0.5. In some cases, mice were mated for a few hours during the day, and the following midnight was considered E0.5. Pregnant females were killed at E8.5 (∼12 somites) or E9 (∼17 somites). *Vangl2*^*Fl/−*^ mice were as previously described (68) and were always phenotypically normal. To obtain *Vangl2*^*−/−*^ embryos, *Vangl2*^*Fl/−*^ stud males were crossed with *Vangl2*^*Fl/−*^ females. *Vangl2*^*Fl/Fl*^ embryos were used as littermate controls. mTmG mice were as previously described (69), and tdTom fluorescence from homozygous mTmG embryos was used for live imaging. *Grhl3*^*Cre/+*^ stud males were as previously described (18) and crossed with mTmG females to lineage trace surface ectoderm cells.

### Immunofluorescence, Image Acquisition, and Analysis.

Embryos were dissected out of their extraembryonic membranes, rinsed in ice-cold phosphate-buffered saline, and fixed in 4% PFA overnight (4 °C). Whole-mount immunostaining and imaging were as previously described (15, 36), and details are provided in *SI Appendix*, *Methods*. Primary antibodies were used in 1:50 to 1:100 dilution and were as follows: rabbit E-cadherin (3195, Cell Signaling Technology), mouse *N*-cadherin (14215S, Cell Signaling Technology), fibronectin (goat SC-6952 [C-20], Santa Cruz Biotechnology and rabbit ab23750, Abcam PLC), rabbit anti-TNS1 (ab233133, Abcam PLC), rabbit anti-laminin (ab11575, Abcam PLC), and rabbit MHC-IIB (909901, BioLegend). For *N*-cadherin staining, antigen retrieval was first performed for 1 h at 100 °C using 10 mM sodium citrate with 0.05% Tween 20, pH 6.0. Secondary antibodies were used in 1:200 dilution and were Alexa Fluor conjugated (Thermo Fisher Scientific). Alexa Fluor 568–conjugated Phalloidin was from Thermo Fisher Scientific (A121380).

### Live Imaging.

Embryos were dissected with an intact yolk sac and transferred into 50% rat serum in Dulbecco’s Modified Eagle Medium. They were then held in place with microsurgical needles (TG140-6 and BV75-3, Ethicon), and a small window was made in the yolk sac and amnion, exposing the HNP. Heartbeat was steady throughout each experiment. Images were captured on Zeiss Examiner LSM 880 confocal (37 °C, 5% CO_2_) using a 20×/NA 1.0 Apochromat dipping objective. X/Y pixels were 0.27 to 0.83 μm, and z step was 1 μm. The time step was 5 to 10 min. Five embryos from independent litters were live imaged for a minimum of 1 h each. Processing of live-imaging movies is described in *SI Appendix*, *Methods*.

### Laser Ablations.

Cable- and tissue-level ablations were performed essentially as previously described (15), and further details are provided in *SI Appendix*, *Methods*.

### Statistical Analysis.

All statistical analysis was performed in OriginPro 2017 (Origin Labs). Individual embryos were the unit of measure. Images are representative of embryos from a minimum of three independent litters. Comparison of two groups was by Student’s *t* test, paired by embryo where appropriate. Comparison of multiple groups was by one-way ANOVA with post hoc Bonferroni. Graphs were made in OriginPro 2017 and are shown either as box plots or as mean ± SEM, in which several embryos were averaged per data point. For box plots, the box shows the 25 to 75th percentiles, and the median is indicated by a line. The whiskers show the 95% confidence intervals, and the outliers are indicated (not excluded).

In Fig. 2 *F* and *G*, distances of the Closure 1 and 2 zippers were normalized against the longest distances recorded for each zipper. Linear regression slopes were estimated using Pearson’s regression and compared by F-test.

### Computational Model.

To model NT closure, we use the vertex model for epithelia (37, 38). Details of model construction, implementation, and parameters are provided in *SI Appendix*, *Methods*.

## Supplementary Material

Supplementary File

Supplementary File

Supplementary File

Supplementary File

Supplementary File

## Data Availability

All study data are included in the article and/or *SI Appendix*. Biological source data are available through a ResearchGate data depository (DOI: 10.13140/RG.2.2.29119.43683) (70). The computer code used in this study is available in the following databases: vertex model simulations for HNP gap closure, GitHub (https://github.com/BanerjeeLab/HNP) and surface subtraction macro courtesy of Dr. Dale Moulding, GitHub (https://github.com/DaleMoulding/Fiji-Macros). Vertex model simulations for HNP gap closure, surface subtraction macro, and biological source data have been deposited in GitHub, ResearchGate (https://github.com/BanerjeeLab/HNP; https://github.com/DaleMoulding/Fiji-Macros; and DOI: 10.13140/RG.2.2.29119.43683). All other study data are included in the article and/or supporting information.
