# Supplementary material for: Hindbrain neuropore tissue geometry determines asymmetric cell-mediated closure dynamics in mouse embryos
Source: Proc Natl Acad Sci U S A. 2021 May 3;118(19):e2023163118. doi: 10.1073/pnas.2023163118 (PMC8126771; doi:10.1073/pnas.2023163118)
Supplement: Supplementary File [file pnas.2023163118.sapp.pdf]

PNAS
www.pnas.org

## Supplementary Information for

# Hindbrain neuropore tissue geometry determines asymmetric cell-mediated closure dynamics in mouse embryos

Eirini Maniou[1], Michael F Staddon[2,3], Abigail Marshall[1], Nicholas DE Greene[1], Andrew J Copp[1], Shiladitya Banerjee[4*], Gabriel L Galea[1,5*]

1: Developmental Biology and Cancer, UCL GOS Institute of Child Health, London, UK

2: Department of Physics & Astronomy, University College London, London, UK

3: Current address: Center for Systems Biology Dresden, Dresden, Germany

4: Department of Physics, Carnegie Mellon University, Pittsburgh, USA

5: Comparative Bioveterinary Sciences, Royal Veterinary College, London, UK

***Corresponding authors:**
4: Carnegie Mellon University, 5000 Forbes Avenue, Pittsburgh, PA 15213, USA
Tel: (+1) 412-773-3728
E-mail: shiladtb@andrew.cmu.edu

1: W2.06, UCL GOS ICH, 30 Guilford St, London, WC1N 1EH, UK
Tel: (+44) 207 905 2326
E-mail: g.galea@ucl.ac.uk

**This PDF file includes:**

- Appendix Methods
- Table S1
- Figures S1 to S9
- Legends for Movies S1 to S4
- Appendix References

**Other supplementary materials for this manuscript include the following:**

- Movies S1 to S4

**Appendix Methods-** Page 3

**Appendix Table S1-** Page 6

**Appendix Figure S1-** Page 7

**Appendix Figure S2**- Page 8

**Appendix Figure S3-** Page 10

**Appendix Figure S4**- Page 11

**Appendix Figure S5**- Page 12

**Appendix Figure S6**- Page 13

**Appendix Figure S7**- Page 14

**Appendix Figure S8**- Page 15

**Appendix Figure S9**- Page 15

**Appendix Movie legends**- Page 16

**Appendix References-** Page 17

## Appendix Methods

### Immunofluorescence, image acquisition and analysis

Images were captured on a Zeiss Examiner LSM 880 confocal using 10 x/NA 0.5 or 20 x/NA 1.0 Plan Apochromat dipping objectives. Whole HNP images were captured with x/y pixel sizes of 0.42-0.59 μm and z-step of 0.8-2.44 μm (speed, 8; bidirectional imaging, 1024×1024 pixels). Images were processed with Zen 2.3 software and visualised as maximum projections in Fiji (1). To visualise the surface ectoderm, the z-stacks were first surface subtracted as previously described (2) to only show the apical 2-3 μm of tissue.

For morphometric analysis, HNP length and width were calculated by annotating the HNP rim and then measuring the major and minor axis using the fit ellipse function in Fiji. To quantify the distance of each zipper from the otic vesicles, reflection images were captured using the 10 x/NA 0.5 dipping objective (633 nm wavelength, x/y pixel size 2.44, z step 3.33 μm). The z stacks were 3D rotated and visualised as maximum projections. For 3D visualisation of reflection images (Fig 2A, E), z-stacks were despeckled in Fiji, filtered with a Kuwahara filter (sampling window width of 5) and opened with the 3D viewer plugin.

### Live imaging

Live imaging datasets were 3D registered in Fiji using the Correct 3D Drift plugin (3). They were then deconvolved using the Richardson-Lucy algorithm (5 iterations) in DeconvolutionLab2 (4). All sequences were surface subtracted (macro above) in order to enable visualisation of surface ectoderm cell borders. Cell migration analysis was done in Fiji using the manual tracking plugin along with the 'chemotaxis and Migration tool' plugin (ibidi). Particle Image Velocimetry (PIV) analysis was performed in Fiji using the in-built Iterative PIV (Cross-correlation) plugin (32 pixel final interrogation window size, normalise median test noise = 1 and threshold = 5). Images were Gaussian-filtered (radius = 2 pixels) before applying PIV.

### Laser ablations

After removal of the extraembryonic membranes, embryos were stained with 1:500 CellMask Deep Red plasma membrane (C10046 Invitrogen) in DMEM at 37$^{o}$C for 5 min. They were

then positioned on agarose plates using microsurgical needles and moved to the microscope stage (heated at 37°C). Tissue-level (5) and cable (6) laser ablations were performed as previously described using a Mai Tai laser (SpectraPhysics Mai Tai eHP DeepSee multiphoton laser).

For cable ablations, a 0.1 μm-wide line was cut using 710 nm wavelength at 100% laser power (0.34 μs pixel dwell time for 10 iterations, 20X/NA 1 Plan Apochromat dipper). One ablation was analysed per embryo. Cable recoil was calculated by measuring the immediate displacement of cell landmarks perpendicular to the ablation.

For tissue-level zippering point ablations, a pre- and post-ablation z-stack was obtained using 10X magnification at 633 nm. Total acquisition time for each stack was ~3 min. The ablations were performed using 800 nm wavelength at 100% laser power (65.94 μs pixel dwell time for 1 iteration, 10X/NA 0.5 Plan Apochromat dipper). The zippering point was ablated using narrow rectangular ROIs, moving sequentially in z to ensure the tissue was ablated.

**Computational Model**

To model neural tube closure, we use the vertex model for epithelia (7, 8). The apical surface of the tissue is modelled by a network of connected edges, with cells described as the polygons and cell-cell junctions as the edges. The tissue mechanical energy given by:

$$E = \sum_\alpha \frac{1}{2} K (A_\alpha - A_0)^2 + \sum_\alpha \frac{1}{2} \Gamma (P_\alpha - P_0)^2,$$

where $\alpha$ indicates the sum over all cells. The first term represents cell area elasticity, with elastic modulus $K$, cell area $A_\alpha$ and preferred area $A_0$. The second term represents a combination cytoskeletal contractility and interfacial adhesion energy, where $\Gamma$ is the contractility, $P_\alpha$ the cell perimeter, and $P_0$ the preferred perimeter. When adhesion dominates over contractility, $P_0$ will be large as cells aim to increase contact length with their neighbours. The mechanical force acting on vertex $i$ is given by $\boldsymbol{F}_i = -\partial E / \partial \boldsymbol{x}_i$, where $\boldsymbol{x}_i$ is the position of the vertex. Assuming that the system is over-damped, the equation of motion is given by:

$$\mu \frac{d\boldsymbol{x}_i}{dt} = \boldsymbol{F}_i,$$

where $\mu$ is the drag coefficient.

To model the closure forces acting on the border cells at the gap, we implement an increased tension $\Lambda_{ps}$ for edges on the gap representing the purse-string (9, 10). The purse-string tension is chosen such that the total tension of the junction is equal to 5 times the mean tissue tension. The tension within the tissue is given by $2\Gamma(P - P_0)$, where $P$ is the mean cell perimeter, since two cells contribute to a single junction, and at the wound edge by $\Gamma(P - P_0) + \Lambda_{ps}$. Thus, we calculate $\Lambda_{ps} = 9\Gamma(P - P_0)$.

Each cell in the tissue may also crawl, with a polarity vector $\boldsymbol{p}_\alpha$ which determines the direction of crawling forces. The crawling applies an additional force to all vertices of that cell, equal to $\mu v_0 \boldsymbol{p}_\alpha$, where $v_0$ is the cell crawl speed. Cells around the gap have a polarity vector equal to the unit vector perpendicular to their edge on the gap (9). Cells within the bulk of the tissue then align polarity with their neighbours, subject to a decay:

$$\frac{\partial \boldsymbol{p}_\alpha}{\partial t} = k_a \sum_\beta (\boldsymbol{p}_\beta - \boldsymbol{p}_\alpha) - k_d \boldsymbol{p}_\alpha,$$

where the sum is over all neighbouring cells indexed by $\beta$, $k_a$ is the polarity alignment rate, and $k_d$ is the polarity decay rate. The resulting effect is that all cells will crawl towards the gap, but with less speed as distance to the gap increases.

**Model implementation**

The model is implemented using Surface Evolver (11). We generate an initial tissue configuration using data from experiments. Given a set of cell centers, and boundary points for the gap and border of the tissue, we generate a Voronoi diagram, giving us the cell shapes. The tissue is then relaxed, with the gap edges fixed to maintain its shape, to a mechanical equilibrium before simulating closure. In experiments, the tissue curves around but is free to deform, thus we use free boundary conditions on the external edges. If an edge shrinks below a critical value, $L_{T1}$, the edge undergoes a rearrangement, or T1 transition, in which a new edge is formed perpendicular to the original junction. The equations are solved numerically by discretizing the equation of motion: $\boldsymbol{x}_i(t + \Delta t) = \boldsymbol{x}_i(t) + \boldsymbol{F}_i/\mu$, where $\Delta t$ is the time step.

**Model parameters**

We non-dimensionalise length by $A_0^{1/2}$ and energy by $K/A_0^2$, giving us a normalized mechanical energy of

$$E = \sum_\alpha \frac{1}{2}(A_\alpha - 1)^2 + \sum_\alpha \frac{1}{2}\Gamma(P_\alpha - P_0)^2.$$

The value of contractility $\Gamma$, and preferred perimeter $P_0$, are chosen to minimise the mean square displacement between the initial vertex positions from experiments and final positions after relaxation to equilibrium. Since we are interested in the percentage of closure time, we use a non-dimensional time by rescaling with the drag coefficient $\mu$, and choose a small time step for numerical stability.

The purse-string tension chosen so that the total tension on the gap edges is five times greater than the mean edge tension, to match experimental recoil rates after laser ablation. The tension on an edge has contributions from the two cells connected to it, giving a mean tension of $2(\bar{P}_\alpha - P_0)$, where $\bar{P}_\alpha$ is the mean cell perimeter. Since the gap has contributions from one cell, the purse-string tension satisfies $\lambda_{ps} + (\bar{P}_\alpha - P_0) = 10(\bar{P}_\alpha - P_0)$. Cell crawl speed is chosen to maintain a constant gap aspect ratio over time.

**Table S1: Final model parameters.**

| **Parameter** | **Symbol** | **Value** |
|---|---|---|
| Contractility | $\Gamma$ | 0.04 |
| Preferred perimeter | $P_0$ | 3.5 |
| Purse-string tension | $\Lambda_{ps}$ | 0.143 |
| Cell crawl speed | $v_0$ | 0.005 |
| Drag coefficient | $\mu$ | 5 |
| Polarity alignment rate | $k_a$ | 0.01 |
| Polarity decay rate | $k_p$ | 0.01 |
| T1 length | $L_{T1}$ | 0.1 |
| Time step | $\Delta t$ | 0.2 |

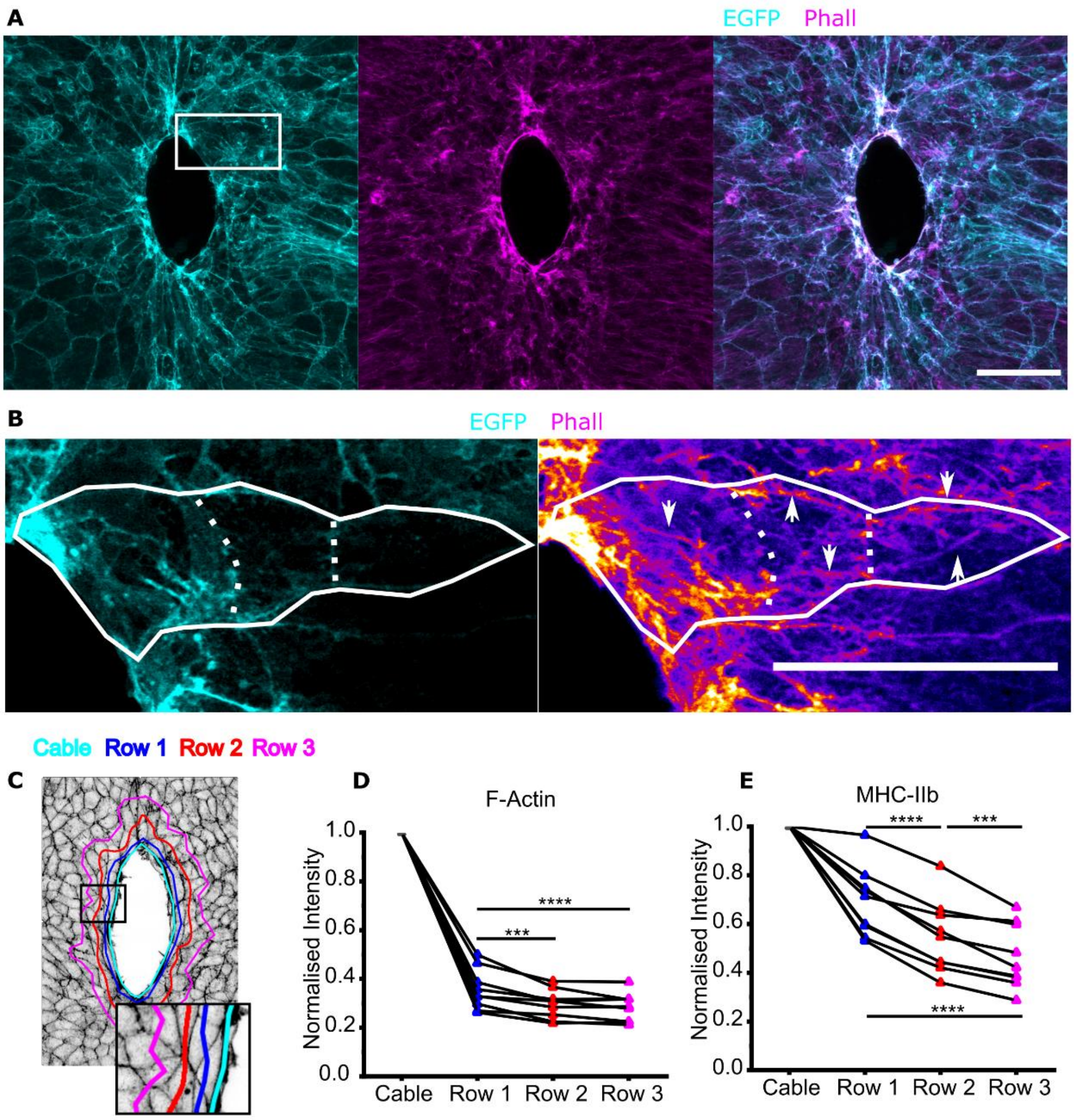


**Appendix Figure S1: Surface ectoderm cells at the HNP edge have enhanced actomyosin levels.**

**A.** Lineage-tracing of surface ectoderm cells (EGFP positive) using Grhl3$^{Cre}$. Scale bar = 50 µm.

**B.** EGFP-labelled cell boundaries from **A** were used to identify F-actin staining within the surface ectoderm cells. Phalloidin staining of F-actin is shown in Fire LUT. Dashed lines indicate the boundaries between three consecutive cells (Rows 1-3). The solid white line outlines the outer borders of the same cells. Arrows indicate linear F-actin arrangements. Scale bar = 25 µm.

**C.** Illustration of how F-actin and MHC-IIb staining intensity was analysed in Row 1-3 cells by selecting a continuous line through adjacent bodies of cells in the same row and comparing intensities within. E-cadherin is shown to identify cell borders.

**D-E.** Quantification of **D)** F-actin and **E)** MHC-IIb staining intensity in Row 1-3 cells, normalised to the staining intensity of the purse-string cables in the same embryo. Points represent individual embryos (n = 8) and lines link points from the same embryo. *** $p < 0.001$ by repeated measures ANOVA with Bonferroni post-hoc.

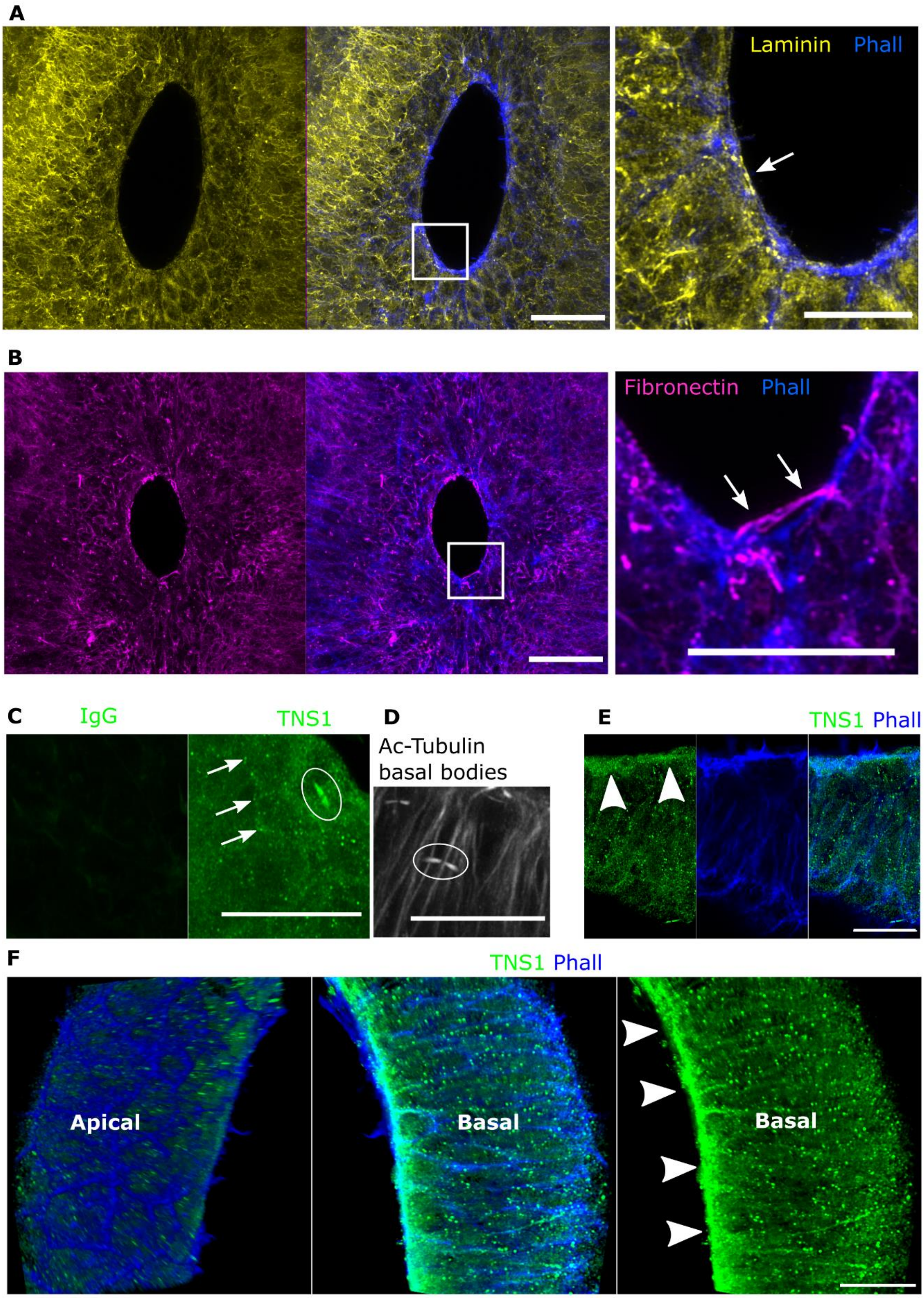


**Appendix Figure S2: ECM adhesion substrates are present at the leading edge of surface ectoderm cells at the HNP rim.**

**A, B.** Laminin (A) and fibronectin (B) immunolocalisation in the HNP of 16 somite stage

embryos. Insets show their localization at the HNP leading edge adjacent to the F-actin purse-string (arrows). Scale bars: full HNP = 50 µm, inset = 25 µm.

**C.** Surface ectoderm immunolocalisation of TNS1 showing its presence in punctate foci resembling focal adhesions (arrows) and basal body-like structures (ellipse). Neither are seen in non-specific IgG control stained and imaged simultaneously. Scale bar = 25 µm

**D.** Acetylated α-tubulin, stained and imaged with the embryos in C, showing localisation to basal bodies (ellipse), but no punctate staining. Scale bar = 25 µm

**E.** Confocal optical slice showing TNS1 puncta present at the F-actin purse-string (arrowheads) along the HNP rim. Scale bar = 25 µm

**F.** 3D reconstruction showing TNS1 accumulation at the leading edge of the surface ectoderm basal surface. C2z is at the top of the image. Scale bar = 25 µm

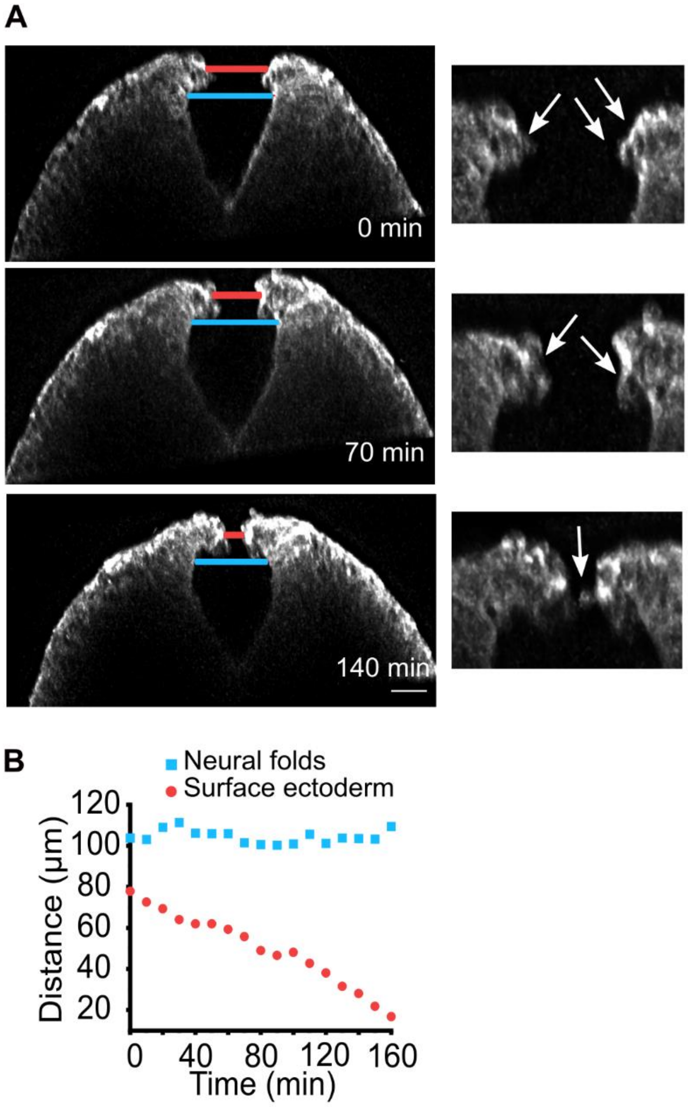


**Appendix Figure S3: Surface ectoderm protrusions lead medial extension during HNP closure.**

**A.** Optically resliced images through a 14 somite stage live-imaged embryo during HNP closure. The red line indicates distance between the surface ectoderm and blue line shows distance between the lateral walls of the neural tube. Arrows in the insets indicate surface ectoderm protrusions. Scale bar = 50 µm.

**B.** Quantification of the distance between the surface ectoderm and neural folds during 160 minutes of live imaging.

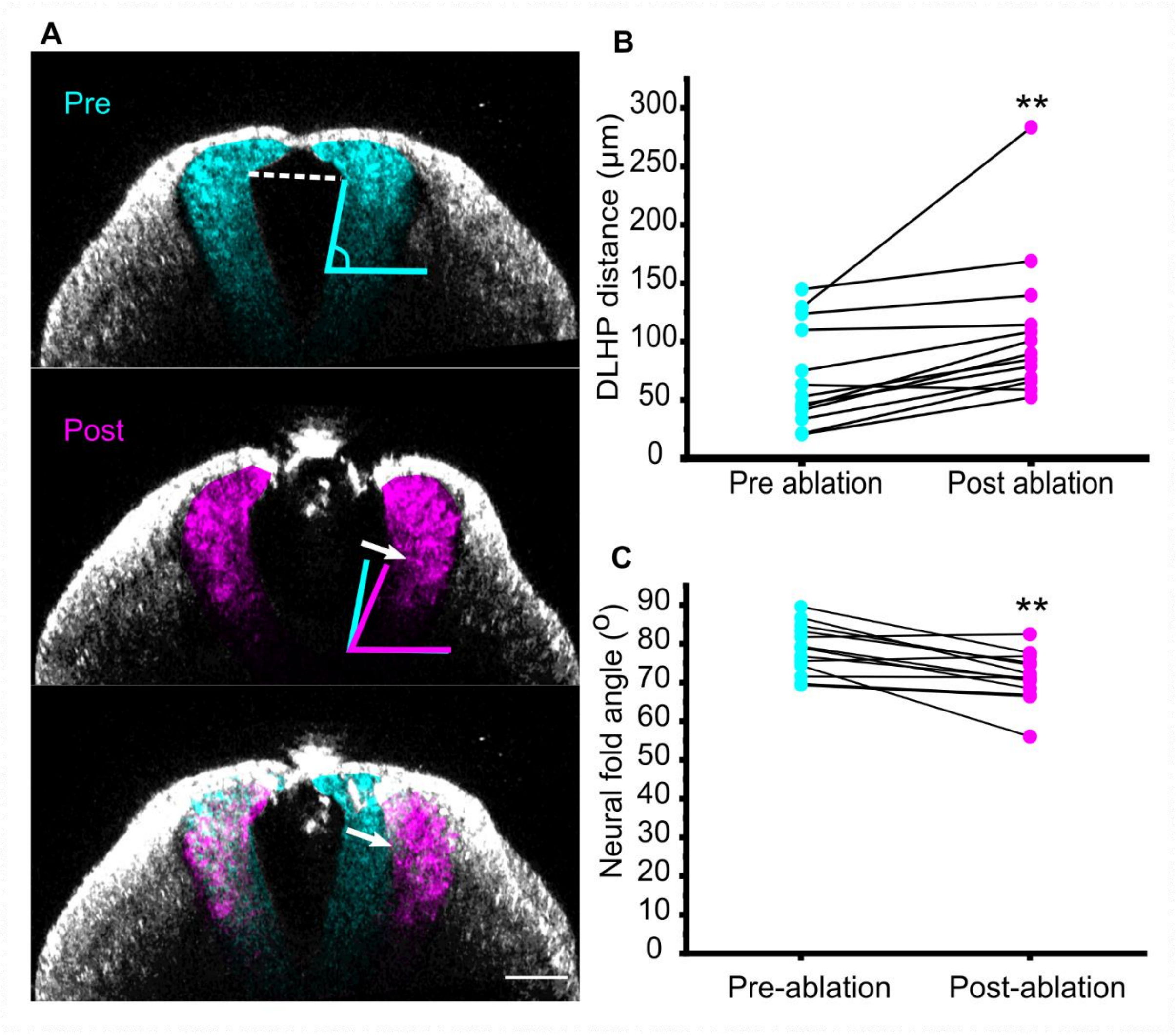


**Appendix Figure S4: The neural folds separate following zippering point ablation.**

**A.** Optically-resliced image showing the position of the neural folds before (cyan) and after (magenta) ablation of the Closure 1 zippering point in an 11 somite stage embryo. The cyan and magenta brackets indicate the calculated angle of the lateral walls of the neural tube. The dashed line indicates the distance between the lateral walls of the neural tube measured at the dorso-lateral hinge points (DLHP). The merged image is registered to the left neural fold. Arrows indicate displacement. Scale bar = 50 µm.

**B-C.** Quantification of the distance between the lateral walls of the neural tube (B) and angle (C) before and after Closure 1 zippering point ablation. Points represent individual embryos and lines link pre- and post-ablation values from the same embryo. N = 13, ** $p < 0.01$ by paired T-test.

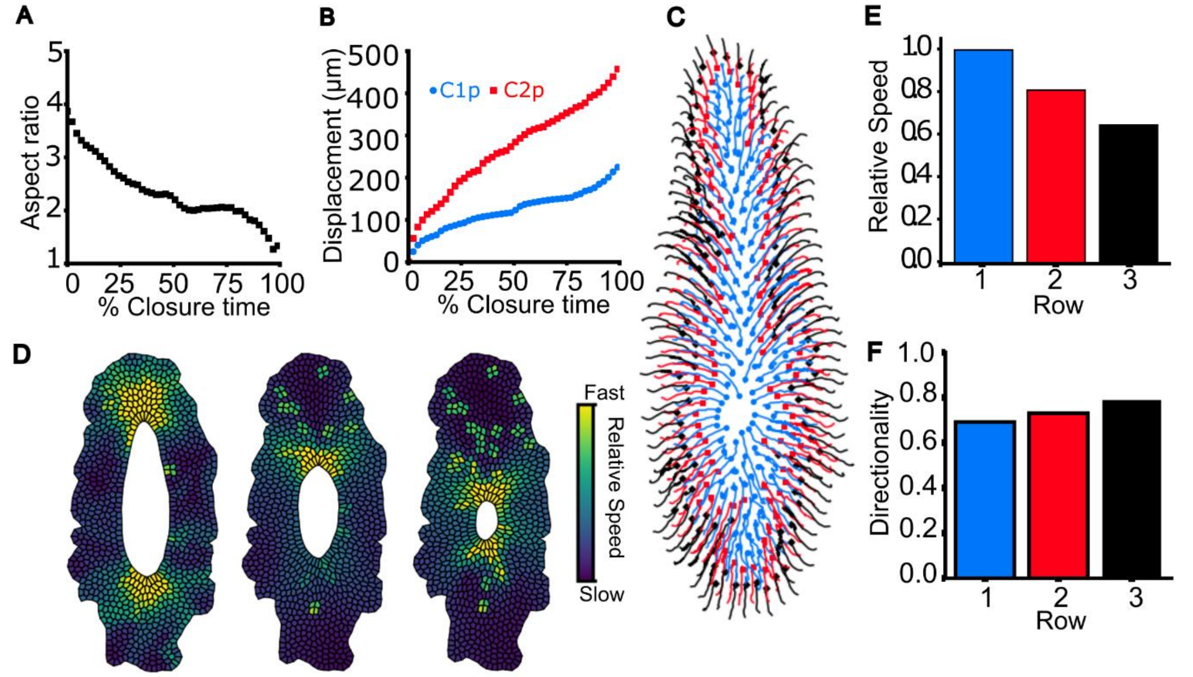


**Appendix Figure S5: Simulations of gap closure with no cell crawling, which produces a round gap.**

**A-B**. Gap aspect ratio (A), and displacement (B) of Closure 1 and 2, against percentage closure time.

**C**. Cell center trajectories during closure. Colour indicates initial cell row.

**D**. Simulation images over time, showing 10%, 50%, and 90% of closure time, from left to right. The cell colour indicates the cell speed. Note that cells along the lateral borders of the HNP display minimal velocity before 50% of closure, after which the gap's aspect ratio drops below the range seen *in vivo* (AR < 2, as shown in **A**).

**E-F**. Mean speed (E), and mean directionality (F) for cells in each of the first three rows around the HNP.

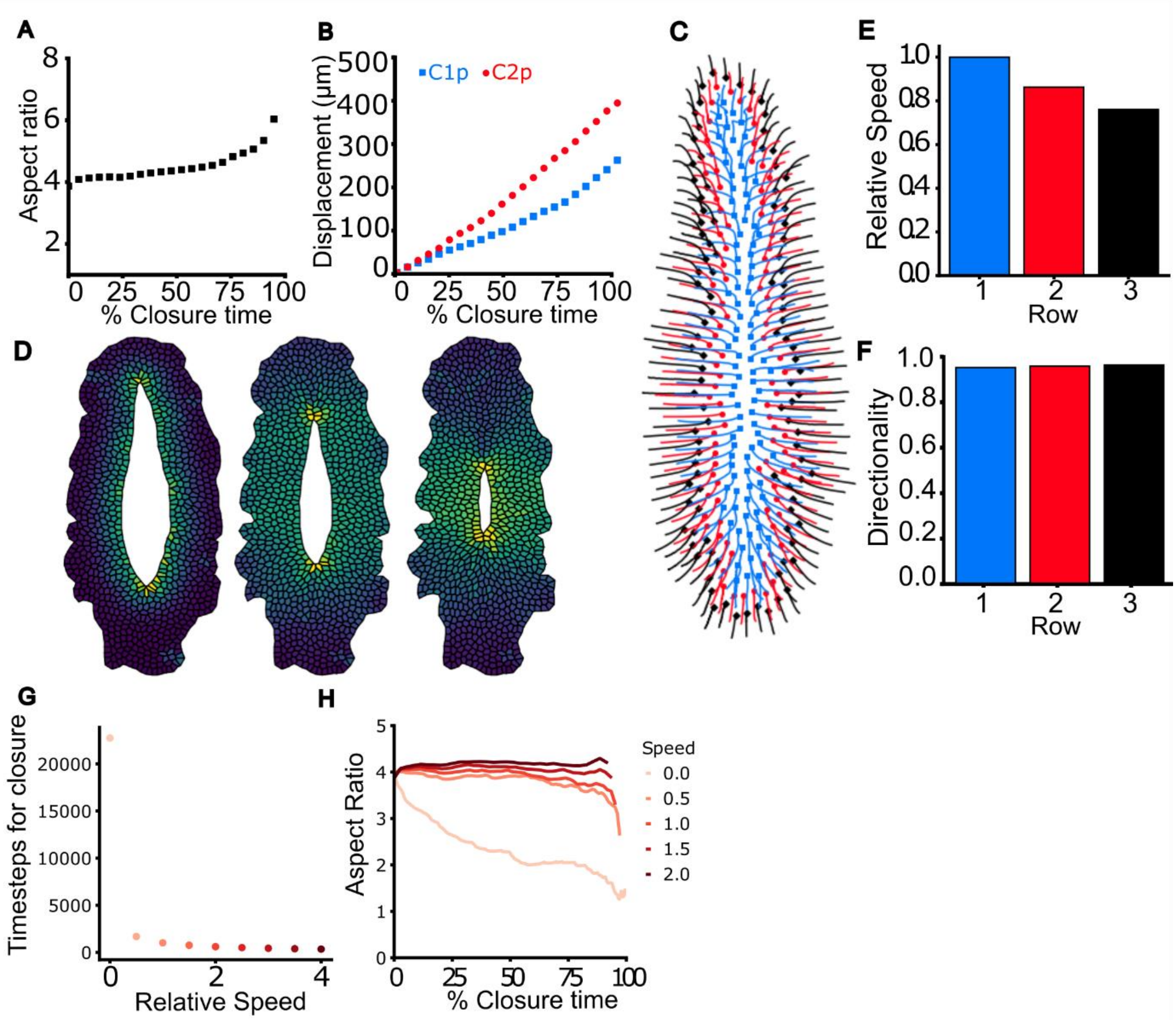


**Appendix Figure S6: Simulation of gap closure with no purse-string, which produces a slit-like gap.**

**A-B**. Gap aspect ratio (A), and displacement (B) of Closure 1 and 2, against percentage closure time.

**C**. Cell center trajectories during closure. Colour indicates initial cell row.

**D**. Simulation images over time, showing 10%, 50%, and 90% of closure time, from left to right. The cell colour indicates the cell speed.

**E-F**. Mean speed (E), and mean directionality (F) for cells in each of the first three rows around the HNP.

**G-H.** Simulation timesteps until closure (G) and aspect ratio against percentage closure time (H), for different cell crawl speeds relative to the default case ($v_0$ = 0.01)

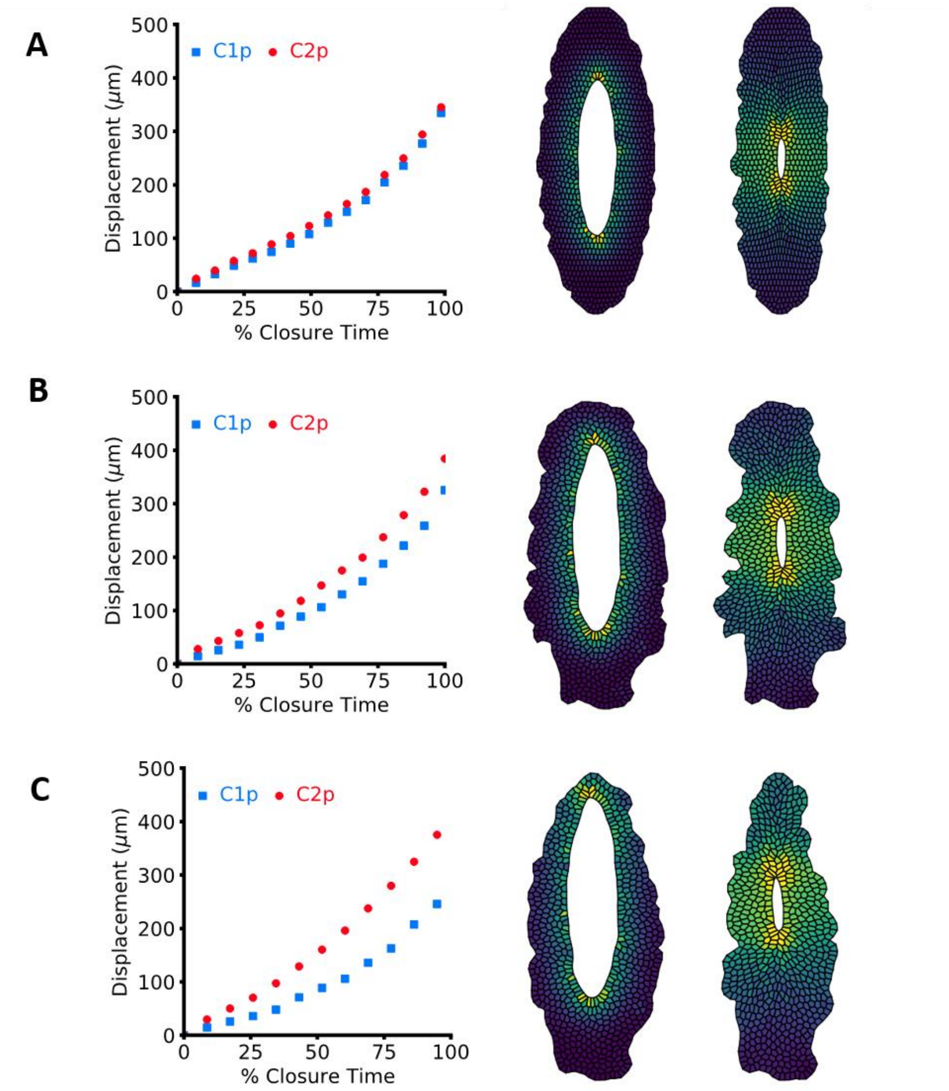


**Appendix Figure S7: Tissue geometry regulates closure rate asymmetry for symmetric gap shapes.**

**A-C**. Displacement of Closure points 1 and 2 plotted against percentage closure time and corresponding simulation snapshots (at 10% and 90% closure time) for symmetric tissue geometry surrounding the gap (A), experimental tissue shape (B), and a highly asymmetric tissue geometry (C).

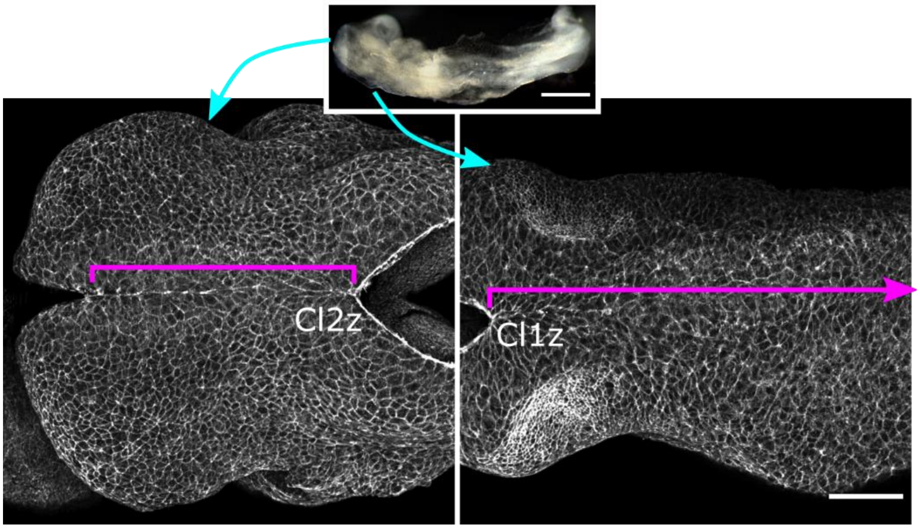


**Appendix Figure S8: There are fewer cells in the plane of closure rostral to Closure 2 than caudal to Closure 1.**

Phalloidin-stained 11 somite embryo showing surface ectoderm cells rostral to the Closure 2 zipper (Cl2z) and caudal to the Closure 1 zipper (Cl1z). The magenta brackets indicates cells in the plane of the Cl2z and Cl1z (continues down the body axis). Scale bar = 100 µm.

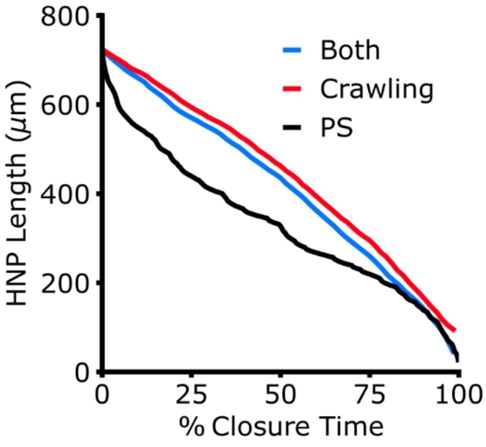


**Appendix Figure S9: Solely purse-string driven closure produces differential closure rates during the closure period.**

HNP length against percentage closure time for simulations with both cell crawling and purse-string, crawling only, and purse-string only.

**Appendix Video Legends**

All animations are provided as GIF images to facilitate visualization on multiple platforms.

**Appendix Movie S1: Live imaging of an E9 mTmG homozygous embryo showing the closing HNP**. tdTom is visualized. C2z is at the top and C1z at the bottom of the image. A z-stack was captured every 10 min for a total time of 170 min. Scale bar: 100 µm.

**Appendix Movie S2: Simulated displacement of cells around the closing HNP with three closure models.** The final combined model, purse string only and crawling only modes of closure are shown. Note that when the purse-string is the only closing force, cells long the lateral borders of the HNP show minimal displacement until the gap becomes rounder. Different colours indicate cells initially in Rows 1-3 and tracks indicate their displacement over time. Each sequence duration is normalized to 100% of closure time.

**Appendix Movie S3: Live imaging of an E9 mTmG homozygous embryo showing the closing HNP.** tdTom is visualised. C2z is at the top and C1z at the bottom of the image. A z-stack was captured every 5 min for a total time of 105 min. Scale bar: 100 µm.

**Appendix Movie S4: Manual tracking of the first three rows of cells around the HNP in Video 3.** Tracks show cells displace in an inward direction along the HNP sides as well as its rostral and caudal ends.

## Appendix References


1. J. Schindelin *et al.*, Fiji: an open-source platform for biological-image analysis. *Nat Methods* **9**, 676-682 (2012).
2. G. L. Galea *et al.*, Vangl2 disruption alters the biomechanics of late spinal neurulation leading to spina bifida in mouse embryos. *Dis Model Mech* **11** (2018).
3. A. Parslow, A. Cardona, R. J. Bryson-Richardson, Sample drift correction following 4D confocal time-lapse imaging. *J Vis Exp* 10.3791/51086 (2014).
4. D. Sage *et al.*, DeconvolutionLab2: An open-source software for deconvolution microscopy. *Methods* **115**, 28-41 (2017).
5. G. L. Galea *et al.*, Biomechanical coupling facilitates spinal neural tube closure in mouse embryos. *Proc Natl Acad Sci U S A* **114**, E5177-E5186 (2017).
6. M. B. Butler *et al.*, Rho kinase-dependent apical constriction counteracts M-phase apical expansion to enable mouse neural tube closure. *J Cell Sci* **132** (2019).
7. R. Farhadifar, J. C. Roper, B. Aigouy, S. Eaton, F. Julicher, The influence of cell mechanics, cell-cell interactions, and proliferation on epithelial packing. *Curr Biol* **17**, 2095-2104 (2007).
8. A. G. Fletcher, M. Osterfield, R. E. Baker, S. Y. Shvartsman, Vertex models of epithelial morphogenesis. *Biophys J* **106**, 2291-2304 (2014).
9. M. F. Staddon *et al.*, Cooperation of dual modes of cell motility promotes epithelial stress relaxation to accelerate wound healing. *PLoS Comput Biol* **14**, e1006502 (2018).
10. R. J. Tetley *et al.*, Tissue Fluidity Promotes Epithelial Wound Healing. *Nat Phys* **15**, 1195-1203 (2019).
11. K. A. Brakke, The surface evolver. *Experiment. Math.* **1**, 141-165 (1992).
