# Supplementary figures and images for: Hindbrain neuropore tissue geometry determines asymmetric cell-mediated closure dynamics in mouse embryos
Source: Proc Natl Acad Sci U S A. 2021 May 3;118(19):e2023163118. doi: 10.1073/pnas.2023163118 (PMC8126771; doi:10.1073/pnas.2023163118)

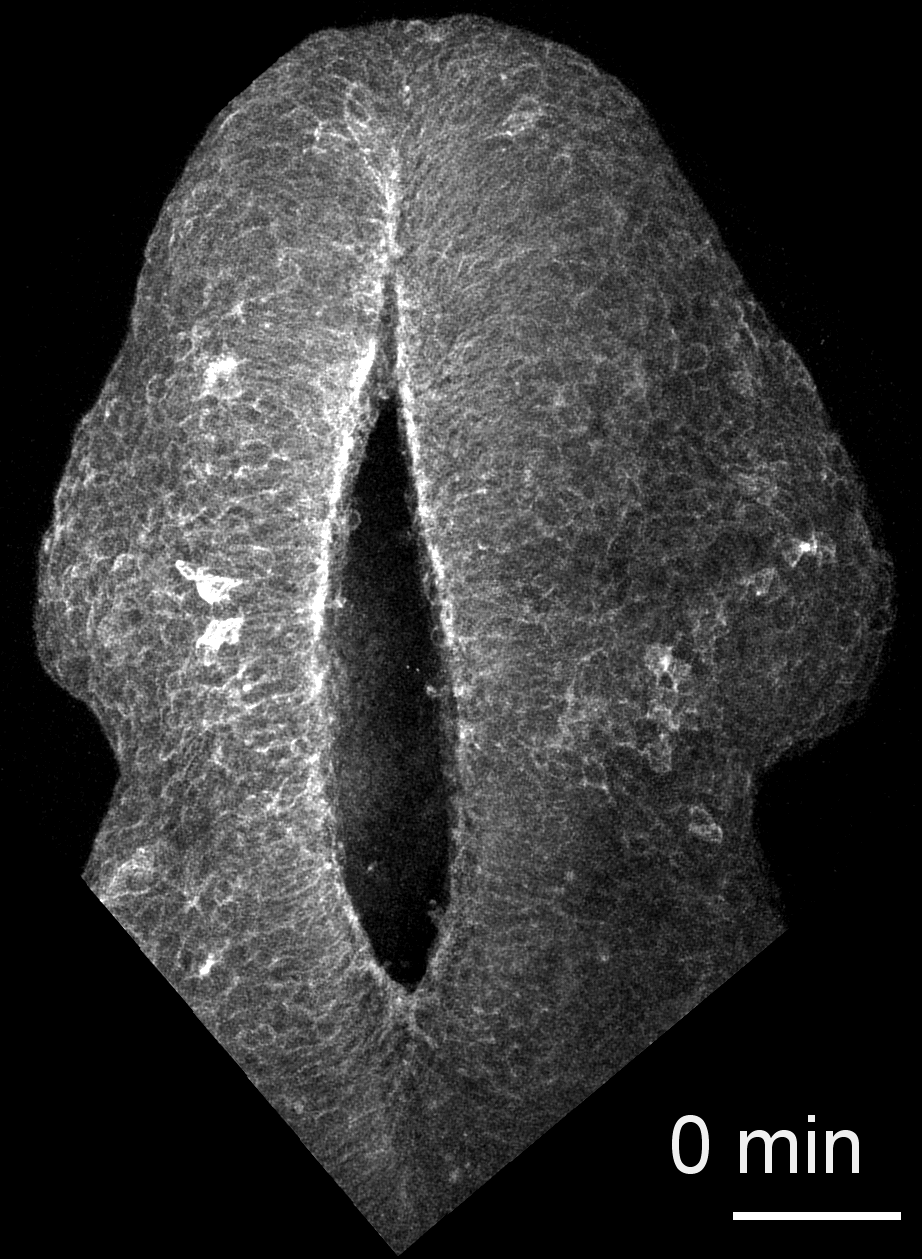

Supplement: Supplementary File [file pnas.2023163118.sm01.gif]

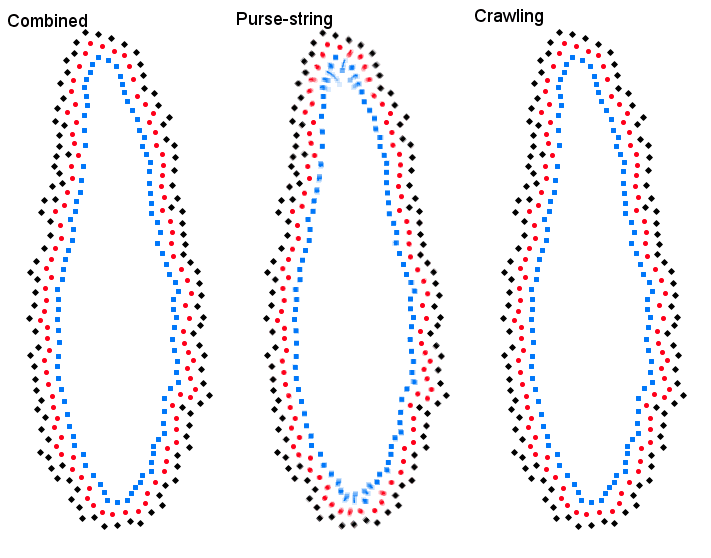

Supplement: Supplementary File [file pnas.2023163118.sm02.gif]

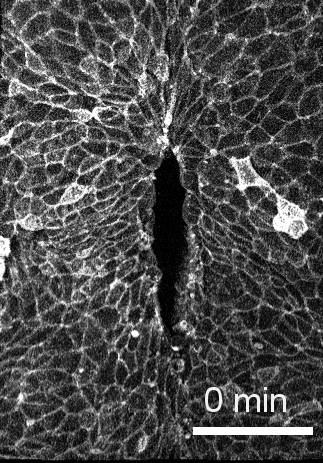

Supplement: Supplementary File [file pnas.2023163118.sm03.gif]

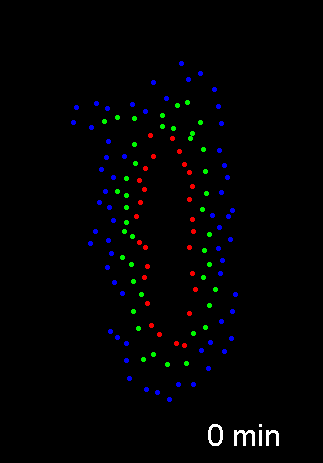

Supplement: Supplementary File [file pnas.2023163118.sm04.gif]
